# Multiomic atlas with functional stratification and developmental dynamics of zebrafish *cis*-regulatory elements

**DOI:** 10.1038/s41588-022-01089-w

**Published:** 2022-07-04

**Authors:** Damir Baranasic, Matthias Hörtenhuber, Piotr J. Balwierz, Tobias Zehnder, Abdul Kadir Mukarram, Chirag Nepal, Csilla Várnai, Yavor Hadzhiev, Ada Jimenez-Gonzalez, Nan Li, Joseph Wragg, Fabio M. D’Orazio, Dorde Relic, Mikhail Pachkov, Noelia Díaz, Benjamín Hernández-Rodríguez, Zelin Chen, Marcus Stoiber, Michaël Dong, Irene Stevens, Samuel E. Ross, Anne Eagle, Ryan Martin, Oluwapelumi Obasaju, Sepand Rastegar, Alison C. McGarvey, Wolfgang Kopp, Emily Chambers, Dennis Wang, Hyejeong R. Kim, Rafael D. Acemel, Silvia Naranjo, Maciej Łapiński, Vanessa Chong, Sinnakaruppan Mathavan, Bernard Peers, Tatjana Sauka-Spengler, Martin Vingron, Piero Carninci, Uwe Ohler, Scott Allen Lacadie, Shawn M. Burgess, Cecilia Winata, Freek van Eeden, Juan M. Vaquerizas, José Luis Gómez-Skarmeta, Daria Onichtchouk, Ben James Brown, Ozren Bogdanovic, Erik van Nimwegen, Monte Westerfield, Fiona C. Wardle, Carsten O. Daub, Boris Lenhard, Ferenc Müller

**Affiliations:** 1 MRC London Institute of Medical Sciences, Du Cane Road, London, W12 0NN, UK; 2 Institute of Clinical Sciences, Faculty of Medicine, Imperial College London, Hammersmith Hospital Campus, Du Cane Road, London, W12 0NN, UK; 3 Department of Biosciences and Nutrition, Karolinska Institutet, NEO, Huddinge SE-141 83, Sweden; 4 Institute of Cancer and Genomic Sciences, Birmingham Centre for Genome Biology, College of Medical and Dental Sciences, University of Birmingham, Vincent Drive, Edgbaston, B15 2TT, Birmingham, UK; 5 Max Planck Institute for Molecular Genetics, Department of Computational Molecular Biology, Berlin, 14195, Germany; 6 Biotech Research and Innovation Centre (BRIC), Department of Health and Medical Sciences, University of Copenhagen, Copenhagen, Denmark; 7 Centre for Computational Biology, University of Birmingham, B15 2FG, Birmingham, UK; 8 Biozentrum, University of Basel and Swiss Institute of Bioinformatics, 4056 Basel, Switzerland; 9 Max Planck Institute for Molecular Biomedicine, Roentgenstrasse 20, Muenster, Germany; 11 Translational and Functional Genomics Branch, National Human Genome Research Institute, Bethesda, MD, USA; 12 Southern Marine Science and Engineering Guangdong Laboratory (Guangzhou), 511458 Guangzhou, China; 13 CAS Key Laboratory of Tropical Marine Bio-Resources and Ecology, South China Sea Institute of Oceanology, Chinese Academy of Sciences, 510301 Guangzhou, China; 14 Environmental Genomics & Systems Biology, Lawrence Berkeley National Laboratory, 717 Potter Street, Suite 164, Berkeley, CA 94710, USA; 15 Genomics and Epigenetics Division, Garvan Institute of Medical Research, Sydney, New South Wales 2010, Australia; 16 Institute of Neuroscience, University of Oregon, Eugene, Oregon, USA; 17 Institute of Biological and Chemical Systems - Biological Information Processing (IBCS-BIP), Karlsruhe Institute of Technology, Karlsruhe, Germany; 18 Max- Delbrück- Center for Molecular Medicine in the Helmholtz Association (MDC), Berlin Institute for Medical Systems Biology (BIMSB), Berlin, Germany; 19 Sheffield Bioinformatics Core, Sheffield Institute of Translational Neuroscience, University of Sheffield, Sheffield, UK; 20 Singapore Institute for Clinical Sciences, Singapore, 117609; 21 Bateson Centre/School of Bioscience, University of Sheffield, Firth Court, Western Bank, Sheffield S10 2TN, UK; 22 Centro Andaluz de Biología del Desarrollo (CABD), CSIC-Universidad Pablo de Olavide-Junta de Andalucía, Seville, Spain; 23 Epigenetics and Sex Development Group, Berlin Institute for Medical Systems Biology, Max-Delbrück Center for Molecular Medicine, 10115 Berlin, Germany; 24 International Institute of Molecular and Cell Biology in Warsaw, Warsaw, Poland; 25 MRC Weatherall Institute of Molecular Medicine, Radcliffe Department of Medicine, University of Oxford, Oxford, UK; 26 Vision Research Foundation, Sankaranethralayas, College Road, Chennai-600006, India; 27 Laboratory of Zebrafish Development and Disease Models (ZDDM), GIGA-R, SART TILMAN, University of Liège, Avenue de l'Hôpital 1, B34, 4000, Liège, Belgium; 28 Laboratory for Transcriptome Technology, RIKEN Center for Integrative Medical Sciences, Yokohama, Kanagawa 230-0045 Japan; 30 Institute of Biology, Humboldt University, 10099 Berlin, Germany; 31 Department of Developmental biology, Signalling research Centers BIOSS and CIBSS, University of Freiburg, 79104, Germany; 32 School of Biotechnology and Biomolecular Sciences, University of New South Wales, Sydney, NSW, Australia; 33 Randall Centre for Cell & Molecular Biophysics, New Hunt’s House, Guy’s Campus, King’s College London, SE1 1UL, UK; 34 Science for Life Laboratory, Tomtebodavägen 23A, Solna SE-171 65, Sweden

## Abstract

Zebrafish, a popular organism for studying embryonic development and for modeling human diseases, has so far lacked a systematic functional annotation program akin to those in other animal models. To address this, we formed the international DANIO-CODE consortium and created a central repository to store and process zebrafish developmental functional genomic data. Our Data Coordination Center (https://danio-code.zfin.org) combines a total of 1,802 sets of unpublished and reanalyzed published genomic data, which we used to improve existing annotations and show its utility in experimental design. We identified over 140,000 *cis*-regulatory elements throughout development, including classes with distinct features dependent on their activity in time and space. We delineated the distinct distance topology and chromatin features between regulatory elements active during zygotic genome activation and those active during organogenesis. Finally, we matched regulatory elements and epigenomic landscapes between zebrafish and mouse and predicted functional relationships between them beyond sequence similarity, thus extending the utility of zebrafish developmental genomics to mammals.

## Introduction

Zebrafish is used as a model vertebrate in over 1,200 laboratories worldwide for studies of organismal, cell and gene function in development, regeneration, behavior, toxicology, and disease modeling. Its popularity is due to its experimental advantages^1^, convenient genetic manipulation tools, wide-ranging genetics resources (e.g. Zebrafish Information Network; ZFIN^2^), and high conservation of disease genes and mechanisms between human and fish ^3^. Use of zebrafish in genomic studies has discovered chromatin signatures^4–6^, DNA codes of promoter usage^7^, regulatory patterns of DNA methylation, and post-transcriptional mRNA regulation^8–13^, while zebrafish single-cell genomics pioneered applications for spatially resolving lineage-specific transcriptomes during development^14^, and comparative genomics has predicted conserved regulatory elements and their long-range target genes^15^. Exploiting the ease of zebrafish transgenesis, automated in vivo imaging and image processing, which can be upscaled to high-throughput^16^, provided validation of predicted disease-associated human enhancers^17,18^. However, despite these many landmark studies, zebrafish has lacked systematic functional annotation programs at a scale seen in other models, including ENCODE^19^, Roadmap Epigenome^20,21^, and modENCODE^22,23^. Thus, disparate zebrafish genomic datasets remain largely inaccessible to thousands of laboratories. Furthermore, while promoters and enhancers from some adult zebrafish tissues have been annotated^24^, embryonic and larval stages lack functional annotation despite representing the bulk of zebrafish-based research. Recognizing these needs, DANIO-CODE was established as a multinational bottom-up effort^25^.

DANIO-CODE aimed to functionally annotate the developing zebrafish genome by i) collecting all published and producing new genomic data from 38 laboratories worldwide and standardizing metadata annotation; ii) creating and maintaining a single data coordination center (DCC) for continued accumulation and user download of zebrafish genomic datasets^26^; iii) developing standardized analysis pipelines and remapping all sequencing datasets; and iv) generating an integrated track hub that allows visualization with common genome browsers. Additionally, DANIO-CODE aimed to conduct an integrated analysis of these datasets to promote discovery, functional element classification and determination of features of developmental dynamics. Finally, in this study we applied novel approaches for comparative analysis of zebrafish and mammalian genomic datasets to uncover conservation of the genomic landscape and to expand the utility of zebrafish developmental genomics resources.

## Results

### The DANIO-CODE Data Coordination Center

**Extended Data Fig. 1:**
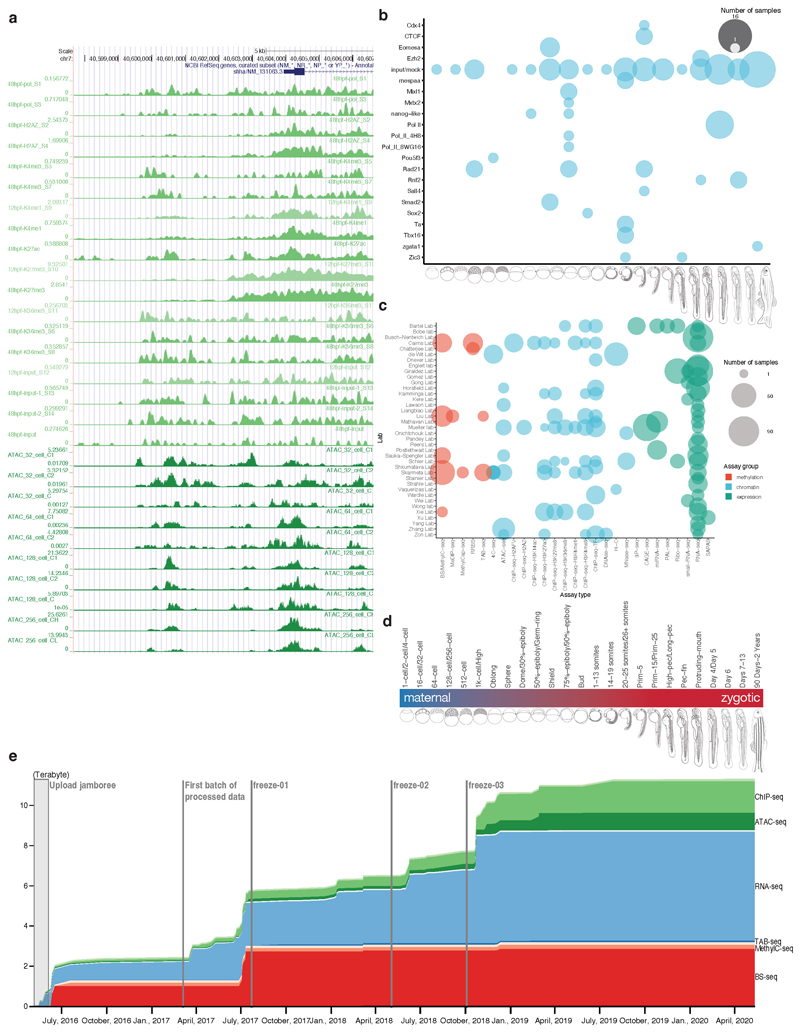
Data increase in the DANIO-CODE Data Coordination Center **a,** Tracks of representative examples of unpublished datasets in a UCSC Genome Browser session including CAGE, ATAC, and ChIP datasets generated by DANIO-CODE laboratories. Promoter region of developmental regulator *shha* gene is shown. **b,** DCC Data availability summary for ChIP with antibodies against Pol II, CTCF and transcription factors as indicated. Stages and stage ranges are indicated on the X axis, the transcription factor occupancy detected is listed on the Y axis. **c,** Data producers and data types matrix indicating the data producer lab (Y axis) and the type of data (X axis). **d,** Data acquisition evolution in the DCC.

**Figure 1:**
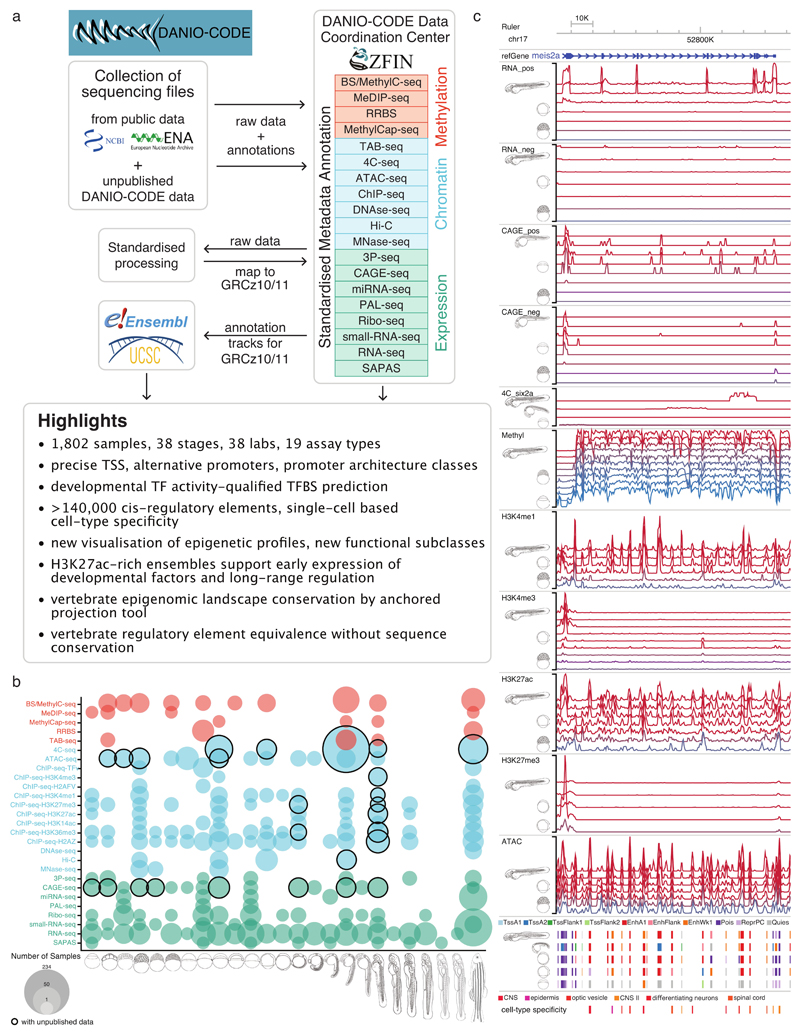
Comprehensive collection and annotation of zebrafish developmental genomic data. **a,** Collection and manual annotation processes of datasets with the DANIO-CODE DCC with highlights of key findings. **b,** Extent of the open repository for developmental multiomic data for zebrafish with assay type (y-axis) and developmental stage (x-axis). Data first reported in this study are highlighted with black circles. **c,** Visualization of temporal dynamics of selected transcriptomic and epigenomic features during development at a developmentally active locus. Coloring of tracks represents developmental series from maternal (blue) to zygotically active stages of embryogenesis (red). Symbols and track colors indicate representative stages (Extended Data Fig. 1d).

We established a Data Coordination Center (DCC) protocol^26^, which we populated with zebrafish developmental genomic data, including standardized annotation of metadata of diverse, often inconsistently annotated published datasets (Fig. 1a), by the DANIO-CODE consortium (https://www.birmingham.ac.uk/generic/danio-code/partners/index.aspx). The DCC is accessible from ZFIN and includes datasets, their underlying samples and sequencing protocols using ZFIN and ENCODE nomenclature (www.danio-code.zfin.org). To identify and analyze the developmental dynamics of genomic features, direct comparison across datasets produced by different laboratories and different protocols is required. To this end, we carried out consistent reprocessing starting from the raw sequencing data (Fig. 1a). Raw sequencing data were collected and reprocessed by standardized pipelines of ENCODE for ChIP-seq and ATAC-seq^27^, FANTOM for CAGE-seq^28^, and producer pipelines for Hi-C, 4C-seq or other data (see Methods). These pipelines are available on GitLab (https://gitlab.com/danio-code). The DCC data include 1,438 published datasets contributed by data producers directly or collected by DANIO-CODE data annotators together with strategically selected datasets for developmental stages from the public domain. In addition, 366 datasets were generated by consortium members to fill gaps and to aid functional annotation and functional element characterization, including 15 CAGE-seq, 18 ChIP-seq, 11 ATAC-seq, 2 Hi-C, and 320 4C-seq datasets (Fig. 1b, Extended Data Fig. 1a,b). Breakdown of the datasets according to data types and stages of development is presented in Fig. 1b. The source of data collection is in Extended Data Figure 1c and Supplementary Table 1. Quality checks and data comparability analyses were carried out for datasets within a data type obtained from multiple laboratories, particularly affecting RNA-seq (Supplementary Fig. 1b), ChIP-seq (Supplementary Fig. 1d-f), CAGE-seq (Supplementary Fig. 2), and ATAC-seq (Supplementary Fig. 1c) data. The DCC continues to be periodically updated (Extended Data Fig. 1e) and is openly accessible to the community for downloading data and uploading new datasets (Video 1 – 2).

The resulting data and reprocessed multiomic datasets represent a comprehensive annotation of the zebrafish genome during normal embryonic development and are available as a public track hub in the UCSC browser and uploadable to the Ensembl genome browser. Figure 1c provides an example developmentally regulated locus covering selected stages visualized by the Washington University Epigenome browser^29^. The tracks further include annotation of approximately 140,000 Predicted ATAC-seq-supported Developmental Regulatory Elements (PADRE) annotated by ChromHMM categories. The bulk sample-based predictions for regulatory elements are complemented with annotations of cell-type specificity of candidate regulatory elements provided by single-cell ATAC-seq^30^ (Video 3 – 5).

### Transcript annotation and core promoter characterization

**Extended Data Fig. 2:**
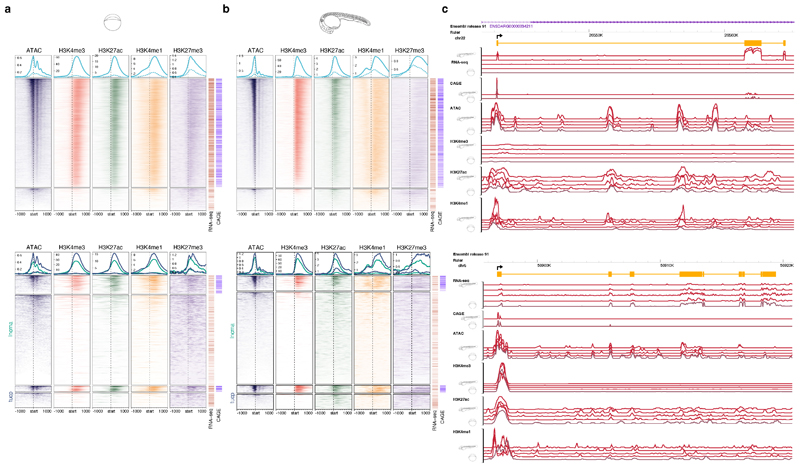
Validation of annotated transcripts **a, b,** Aggregation plots and heatmaps of open chromatin and epigenetic features of annotated transcripts and CAGE-seq validation of TSSs (bars on the right of each panel) are shown for the Dome **(a)** and Prim-5 **(b)** stages. Top panels show protein coding genes (n=14,471, of which 12,031 are supported by CAGE for the Dome stage; n=16,478, of which 13,769 are supported by CAGE for the Prim-5 stage) and bottom panels show lncRNA (n=1,780, of which 302 are supported by CAGE for the Dome stage; n=1,551, of which 220 are supported by CAGE for prim-5) and TUCP (n=336, of which 97 are supported by CAGE for the Dome stage; n=329, of which 112 are supported by CAGE for the Prim-5 stage) genes **c,** Example screenshot of novel lncRNA(top), and novel TUCP (bottom) transcripts and associated epigenomic features.

**Figure 2:**
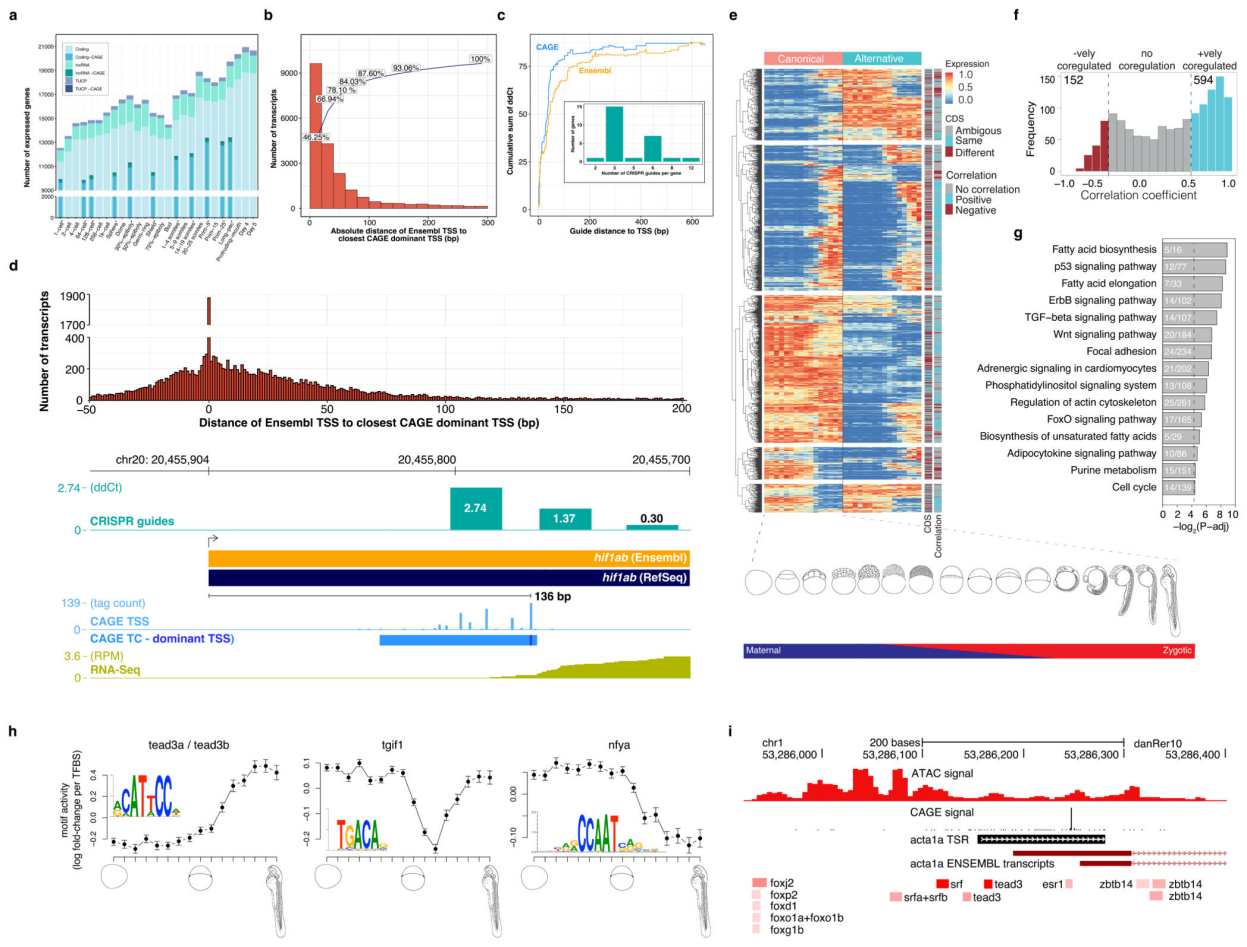
Transcript categories and single-nucleotide resolution 5’ end verification during development **a,** DANIO-CODE transcript 5’ ends supported by CAGE TSS during stages of development. **b,** Distribution of absolute distance of Ensembl TSSs to CAGE dominant TSSs in the Prim-5 stage. **c,** Relationship between guide distance to TSSs and ddCt. Insert: number of dCas guides for all 26 tested genes. **d,** CAGE defined TSSs increase the accuracy of promoter identification and support dCas inhibition guide reagent designs. Distance between Ensembl TSSs and CAGE dominant TSSs (top). Genome view with CRISPR guide position and efficacy, Ensembl and RefSeq transcripts, CAGE and RNA-seq expression (bottom panel). **e,** Heatmap shows the dynamics of expression levels of reference and alternative promoters across 16 developmental stages represented as images. Expression levels are scaled in the range of 0-1 for each row. Reference and alternative transcripts using same and different coding sequence (CDS) start are denoted. Transcript pairs without full CDS annotation are denoted as ambiguous. **f,** Distribution of correlation coefficient of expression levels of promoters across 16 developmental stages. **g,** Enrichment of KEGG pathways on multi promoter genes. The adjusted *P* value cut-off is 0.05, denoted by a vertical dashed line. The number of genes in KEGG pathways and those overlapping with multi promoter genes is shown inside the bars, **h,** MARA motif activity plots of 3 TF motifs across development. Posterior means and standard deviations(depicted as error bars) are based on analysis of the expression levels of all n = 27,781 promoters for each sample. Motif logos are depicted as insets. **i,** Genome browser view of the *actin alpha 1a* promoter. From the top: ATAC-signal, CAGE signal, a single TSR (black), two Ensembl transcripts (dark red), and TFBSs predicted to regulate this TSR (red) are shown. Color intensities of the TFBSs reflect MARA scores of predicted regulatory role of TFs.

As genome-wide transcriptome analyses^31–34^ fail to annotate 5' UTRs precisely, we used DANIO-CODE expression data to improve current Ensembl models of developmentally active genes. We utilized 139 developmental RNA-seq samples to identify 31,458 genes comprising 55,596 transcripts (Fig. 2a, Supplementary Table 2), among them 167 novel transcripts of uncertain coding potential (TUCP) and 726 lncRNA genes not previously annotated by Ensembl and supported by CAGE signals (Extended Data Fig. 2, Supplementary Table 3). We mapped 5’ transcription start sites (TSSs) from 34 CAGE samples in 16 developmental stages (Fig. 2a). We applied promoter-calling criteria to CAGE data (Methods, Supplementary Fig. 2a-c) resulting in 22,500 active promoters per CAGE sample on average, corresponding to 16,303 genes (Supplementary Table 4), adding 4,070 novel promoters to 18,461 previously annotated Ensembl TSSs (GRCz10). To supplement the promoterome with *cis*-regulatory sites, we curated 581 regulatory motifs representing 814 zebrafish transcription factor (TFs), and predicted binding sites for these motifs across all promoters (Methods).

**Extended Data Fig. 3:**
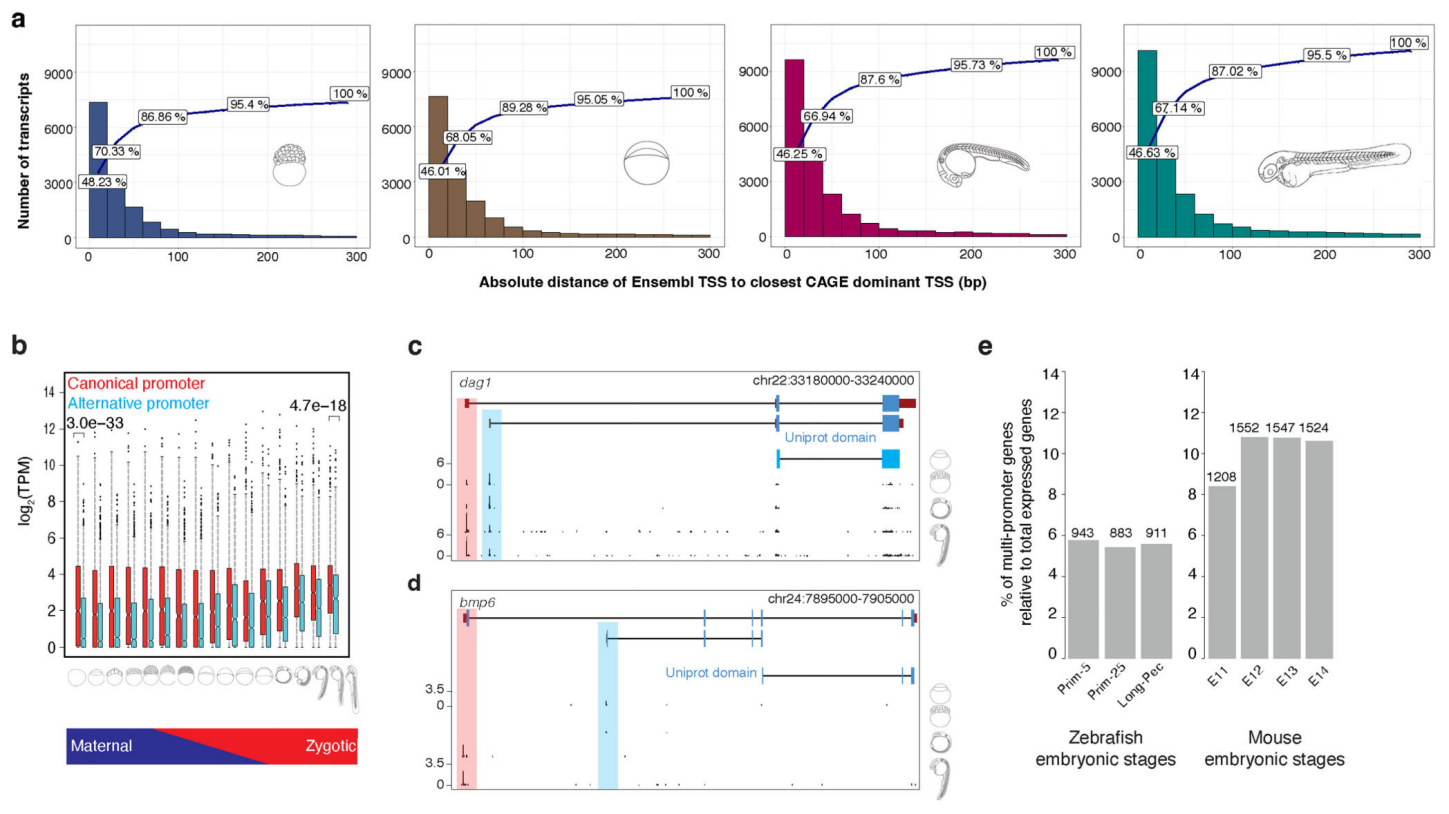
Characterisation of promoter calling precision and alternative promoter usage in annotated transcripts **a,** Frequency distribution of Ensemble transcripts 5’ ends binned according to distance (bp) from CAGE dominant peak as indicated on X axis. Cumulative frequency depicted by line. Developmental stages are indicated by embryo symbols. **b,** Box plot shows the expression levels of canonical and alternative promoters across 16 developmental stages. P-values denote the significant difference in expression levels between canonical and alternative promoters during two stages at fertilized-egg (P=3.0E-33; t-test two-sided) and long pec (P=4.7E-18; t-test two-sided). **c,** A UCSC browser screenshot of the gene dag1 shows the alternative promoter (highlighted in cyan) is upstream of the start codon (pointed by arrow), thus altering only 5’UTR but not protein. The numbers on the y-axis represent the normalized tags per million (TPM) of CAGE tags. The Uniprot domain track denotes the annotated protein domain in the Uniprot database. **d,** A UCSC browser screenshot of the gene bmp6 shows the alternative promoter (highlighted in cyan) is downstream of the start codon (pointed by arrow) and alters the N terminal of the protein. **e,** Bar plots show the fraction of multi-promoter genes relative to the total expressed genes in zebrafish and mouse embryonic stages. The numbers on top of bar plot represent the actual number of multi-promoter genes. E11 represents embryonic day 11 and so on for E12, E13 and E14.

Our above definition of promoters at single-nucleotide resolution may offer important guidance for promoter-targeted gene manipulation. For instance, gene promoter targeting for transcription block may be useful in reverse genetic experiments to avoid mutant RNA-mediated genetic compensation, which may mask mutant phenotypes and hinder dissection of gene function^35^. We compared Ensembl’s RNA-seq-based TSS with our CAGE-seq-based TSS and found a substantial discrepancy in position (Fig. 2b, Extended Data Fig. 3a), potentially impacting guide-RNA design for CRISPR/Cas targeting. Multiple dCas guide positions were designed and their impact on expression reduction with increased distance between the guide target and dominant CAGE-defined TSS was tested. Efficiency of dCas inhibition was higher when CAGE-dominant, compared to Ensembl, start sites were used (Fig. 2c,d, Supplementary Table 5), demonstrating the importance of accurate TSS detection and the improved accuracy of CAGE over current Ensembl pipeline in promoter detection.

Using these data we identified 1,293 multi-promoter genes (Supplementary Table 6), where 1,176 genes had one reference and one alternative promoter and 117 genes had two or more alternative promoters. Correlation of expression levels of reference and alternative promoter pairs indicated both convergent (cyan in Fig. 2e, f) and divergent (brown) dynamics during development. The expression of reference promoters was on average higher than those of alternative promoters (Extended Data Fig. 3b). Among 978 transcript pairs with full-length coding sequence (CDS) annotation, 373 (38%) of the alternative promoters affected only the 5’ UTR (e.g., *dag1*, Extended Data Fig. 3c), whereas the remaining 605 altered the N-terminal protein sequence (e.g., *bmp6*, Extended Data Fig. 3d). We analyzed mouse CAGE-seq^28^ data from comparable embryonic stages and annotated 1,779 multi-promoter genes (Extended Data Fig. 3e, Supplementary Table 7). About one third (294; 30%) of identified mouse orthologs of zebrafish multi-promoter genes (974; 75%) utilized alternative promoters. Orthologs of multi-promoter genes were likely (*P* = 2.7 × 10^-5^; Fisher’s exact test) to be expressed in similar stages and highly likely (*P* = 3.24 × 10^-58^; Fisher’s exact test) to have multiple promoters in mouse. Multi-promoter genes were enriched in KEGG signaling pathways in zebrafish (Fig. 2g) and mouse (Supplementary Table 8), suggesting vertebrate conservation of alternative promoters in signal transduction-associated genes.

**Extended Data Fig. 4:**
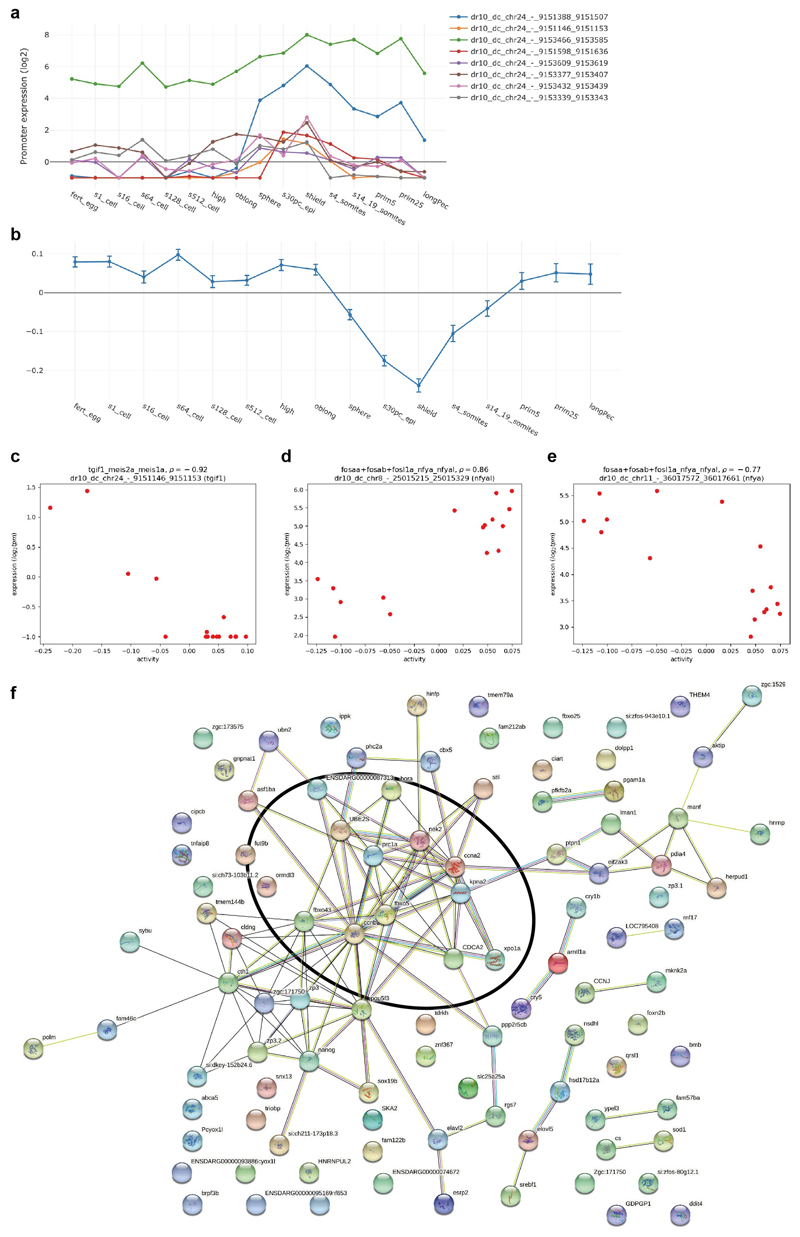
Motif activity analysis MARA predicts up-regulation of Tead3’s activity from gastrulation onwards (Figure 2). For each potential target promoter with Tead3 binding site, MARA quantifies the extent the Tead3 motif activity explains the target’s expression dynamics (log-likelihood score). For each GO category the sum of log-likelihoods for all genes in the category was calculated. Suppl. Table 13 shows the GO biological process categories with the highest total log-likelihoods. Top categories correspond to processes in which Hippo signalling during early development in zebrafish has been implicated. a,b, The *tgif1* promoters are transiently upregulated during gastrulation **(a)** while the targets of Tgif1 are transiently down-regulated **(b),** supporting Tgif1 as a repressor. Posterior means and standard deviations (depicted as error bars) are based on analysis of the expression levels of all n= 27781 promoters for each sample. **c,** Scatterplot of TGIF1_MEIS1a_MEIS2a motif activity (horizontal axis) against total *tgif1* mRNA expression (vertical axis) shows motif activity and TF expression are highly anti-correlated (Pearson correlation -0.92). **d,** Scatterplot of the FOS/NF-Y motif activity (horizontal axis) against expression of the *nfyal* gene shows positive correlation (Pearson correlation coefficient 0.86). **e,** Scatterplot of the FOS/NF-Y motif activity (horizontal axis) against expression of the *nfya* gene shows negative correlation (Pearson correlation -0.77). As shown in Figure 2h, MARA predicts that targets of NF-Y are down-regulated from the sphere stage onwards, thus as the NF-Y motif activity decreases during development. The expression of *nfya* is up whereas *nfyal* is down-regulated, suggesting that Nfya may replace Nfyal in the NF-Y complex. **f,** STRING database network picture of the predicted target genes of the NF-Y motif. The black oval indicates a set of target genes involved in mitosis and G2/M transition, consistent with the documented role of NF-Y.

Precision promoter annotation and expression dynamics allow exploitation of this resource to predict TF activity regulating the promoters. We implemented Motif Activity Response Analysis^36,37^ for zebrafish. MARA models promoter expression dynamics in terms of the annotated TF binding sites, to infer which TFs most significantly drive expression changes during development. Figure 2h shows the inferred activity profiles of three TFs with strong effects on genome-wide expression patterns. While Tead3 targets are upregulated from gastrulation onwards, Tgif1 targets are transiently downregulated, and NF-Y targets are down-regulated from the sphere stage onwards, consistent with the known activities of these TFs^38–42^ (Extended Data Fig. 4, Supplementary Table 9). MARA predicts significantly changing regulatory activities for regulatory motifs and assigns candidate regulator TFs to promoters (Fig. 2i). We have integrated our zebrafish annotations into the ISMARA webserver (ismara.unibas.ch) to allow this activity analysis on any RNA-seq data.

### Classification of genomic regulatory regions in development

**Extended Data Fig. 5:**
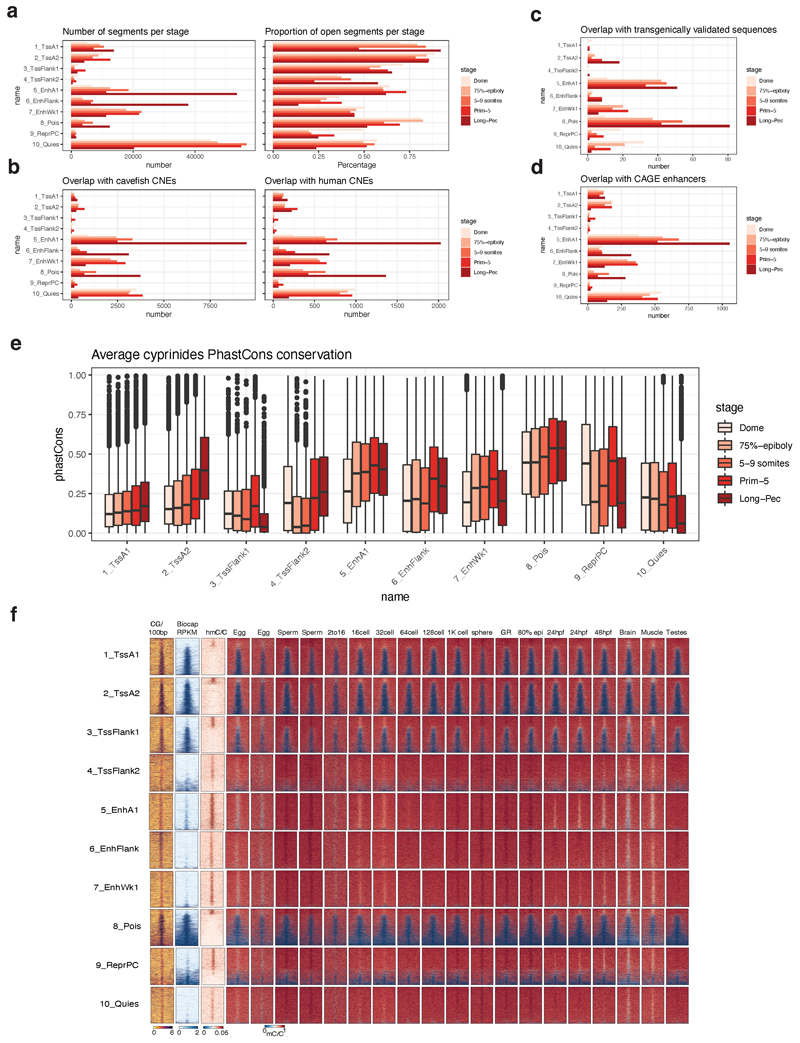
PADREs validation **a,** Left: Number of PADREs assigned to each chromatin state for every developmental stage. Right: Proportion of ChromHMM states present in PADREs for each stage. **b,** Number of annotated PADREs overlapping Mexican cavefish (left) and human (right) CNEs for each stage. **c,** Number of annotated PADREs overlapping transgenically validated enhancers for each stage. **d,** Number of annotated PADREs overlapping CAGE-defined eRNAs for each stage. **e,** phastCons scores distribution of annotated PADREs for each stage. **f,** Methylation profile throughout the development of annotated PADREs at the Prim-5 stage.

**Figure 3:**
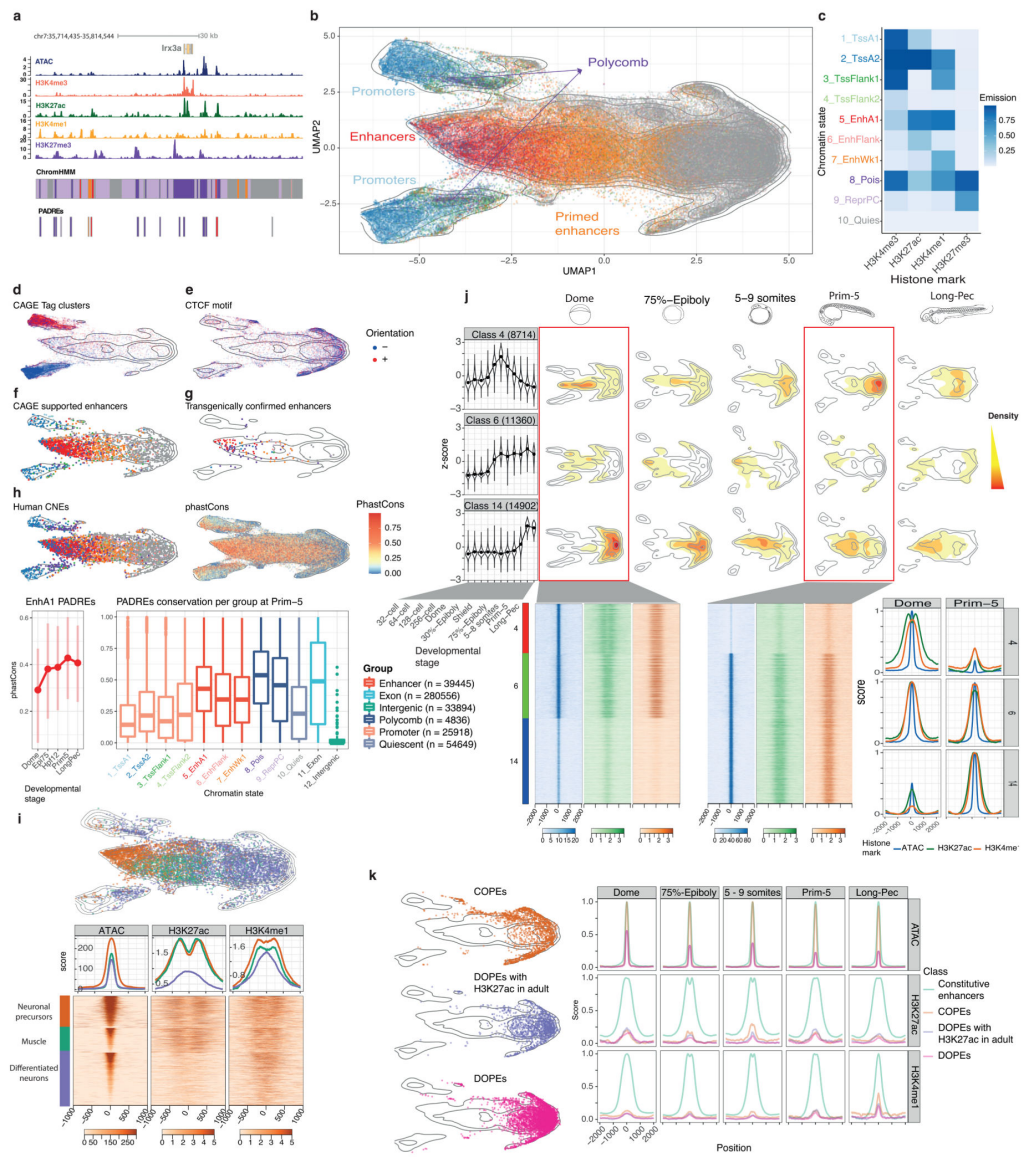
Classification of developmental *cis*-regulatory elements. **a,** Genome browser screenshot showing ChromHMM classification of PADREs, and respective histone post-translational modification signals used to define them. **b,** UMAP plot of PADREs at the Prim-5 stage. Each point represents one open chromatin region, colored by functional assignment. **c.** Occurrence probabilities of chromatin marks for ChromHMM states. The states function was manually assigned using The Roadmap Epigenomic annotations as reference. 1_TssA1, 2_TssA2 = Active TSS, 3_TssFlank1, 4_TssFlank2 = TSS flanking region, 5_EnhA1 = Active enhancer, 6_EnhFlank = Enhancer flanking region, 7_EnhWk1 = Primed enhancer, 8_Pois = Poised elements, 9_PcRep = Polycomb repressed regions, 10_Quies = Quiescent state. **d-g.** UMAP plot showing PADREs overlapping with CAGE promoters **(d),** CTCF motif **(e),** eRNA enhancers **(f),** and transgenically validated enhancers **(g).** The transgenically validated enhancers are predominantly associated with enhancer associated chromatin states (Supplementary Table 11). **h,** UMAP plot showing the mean phastCons score for each PADRE (top right) and overlap with human CNEs (top left). The bottom sub-panel shows the distribution of phastCons scores of active enhancers throughout development (left, bars represents inter-quartile range), as well as the distribution of the phastCons score for PADREs separated by function at the Prim-5 stage. Two-sided Wilcoxon Rank Sum Test was used to calculate *P* values between promoters and enhancers (*P* = 2.2 × 10^-16^) and enhancers and Polycomb-associated elements (*P* = 2.2 × 10^-16^). Exons and intergenic regions were added as reference (right) **i,** Top: Position of cell-type specific elements on the UMAP plot. Bottom: ATAC, H3K27ac, and H3K4me1 signals around the peak summit of cell-type specific PADREs. **j,** Upper: Openness profile of selected SOM classes (4: early, 6: post-ZGA constitutive, and 14: late class), and their position density on the UMAP plots of different developmental stages. Bottom: Heatmap of signal intensity of ATAC, H3K27ac, and H3K4me1 at the Dome and the Prim-5 stages, along with their respective profiles. **k,** left: Position of COPEs, DOPEs, and DOPEs marked with H3K27ac in adult tissues on the UMAP plot. Right: Profiles of ATAC, H3K27ac, and H3K4me1 of COPEs, DOPEs, DOPEs marked in adult tissues, and other constitutive elements throughout development.

Next, we aimed to generate a comprehensive atlas of zebrafish developmental regulatory elements. We defined reproducible ATAC-seq^43^ peaks as Predicted ATAC-seq-supported Developmental Regulatory Elements (PADREs) in 4 pre-zygotic genome activation (ZGA) and 7 post-ZGA stages, which we further classified based on the presence of four histone marks using ChromHMM^44,45^ in 5 post-ZGA stages (Fig. 3a, Supplementary Fig. 3, Extended Data Fig. 5a).

**Extended Data Fig. 6:**
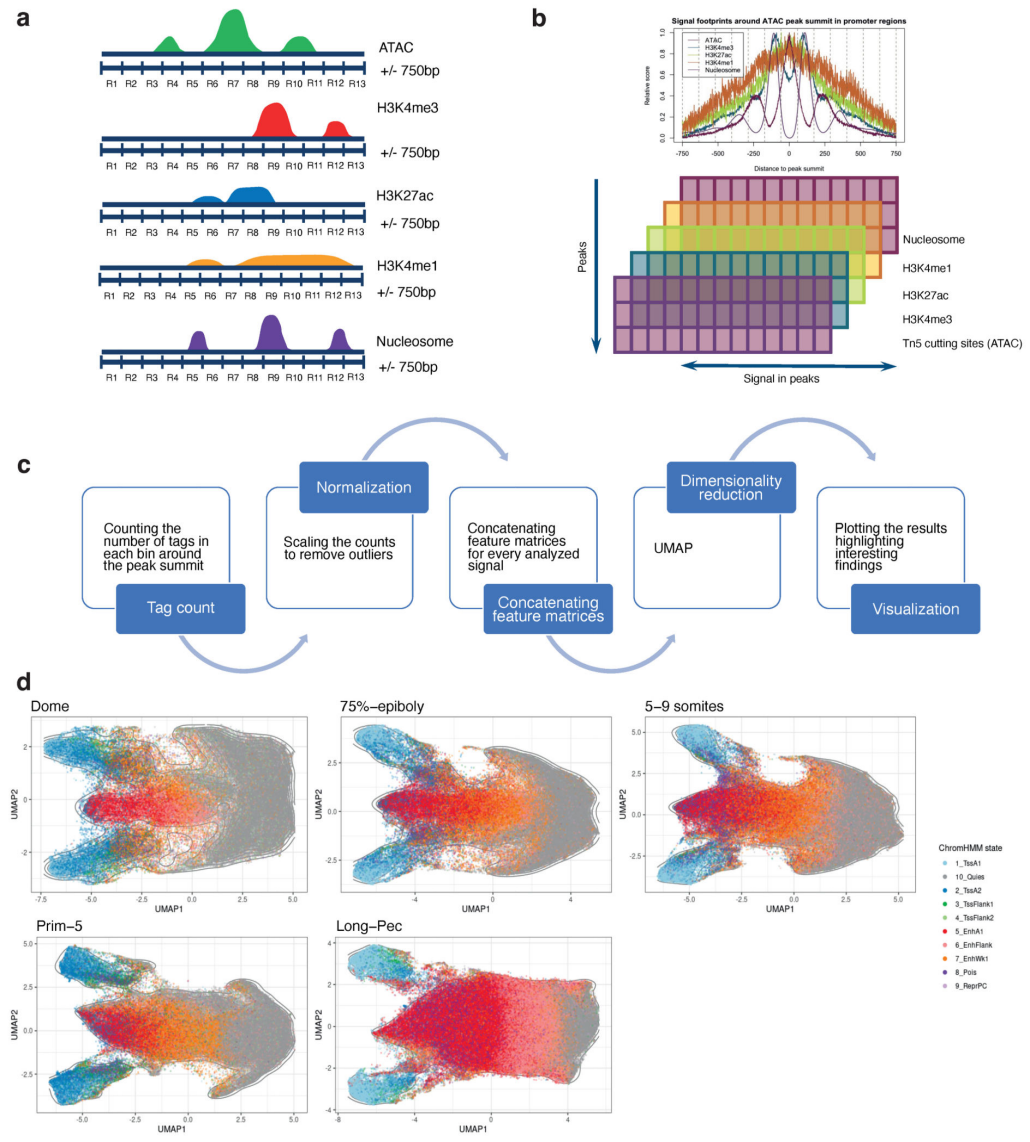
UMAP visualisation of regulatory elements **a-c,** Schematic representation of UMAP visualization of PADREs (for details, see Methods). R1-13 represent bins used to make the model. **d,** UMAP plot of annotated PADREs for each developmental stage analysed.

To examine the developmental dynamics of PADREs, we developed a UMAP-based method (Methods, Extended Data Fig. 6a-c) that can identify known functional classes and potentially novel subclasses during development. The UMAP plot of PADREs (Fig. 3b, Extended Data Fig. 6d) separated most ChromHMM-derived functional classes, including promoters from enhancers (Fig. 3c). Near-symmetry around the y-axis reflects strand directionality and was most prominent among promoters (Fig. 3d). Two prominent clusters, that stretched upward and downward from the right apex and bear no chromatin marks are enriched for the CTCF motif with well-positioned flanking nucleosomes^46^ (Fig. 3e, Supplementary Fig. 4). Enhancer predictions were validated with two independent sets: 1) enhancers with bidirectional eRNA signals^47^ called from nuclear CAGE; and 2) a manually curated catalog of published enhancers functionally validated in transgenic reporter assays (Supplementary Table 10). Both co-localized with enhancer-classified PADREs on the UMAP (Fig. 3f,g, Extended Data Fig. 5c, d), demonstrating the utility of the method. DNA methylation analysis revealed CG-rich, promoter-associated PADREs persistently hypomethylated across stages, and less CG-dense enhancer-associated PADREs gradually hypermethylated during development before becoming hypomethylated in adult somatic tissue. Dynamically methylated PADREs varied in the onset and degree of hyper/hypomethylation: e.g., conserved phylotypic enhancers^11^ commenced hypomethylation at the Prim-5 stage (Extended Data Fig. 5f).

Next, we assessed the evolutionary conservation of PADREs by overlapping with human Conserved Non-Coding Elements (CNEs) and calculating the phastCons score for each PADRE (Fig. 3h, top, Extended Data Fig. 5b). Early-acting enhancers appear less conserved than those activated later (Fig. 3h, bottom left; Extended Data Fig. 5e). phastCons scores of enhancers were higher on average than promoters (Fig. 3h, bottom right). Poised elements were the most conserved, suggesting that Polycomb-bound enhancers are a specific class critical for differentiation and organogenesis^17,48^, and contributing to the hourglass model of development^49^.

**Extended Data Fig. 7:**
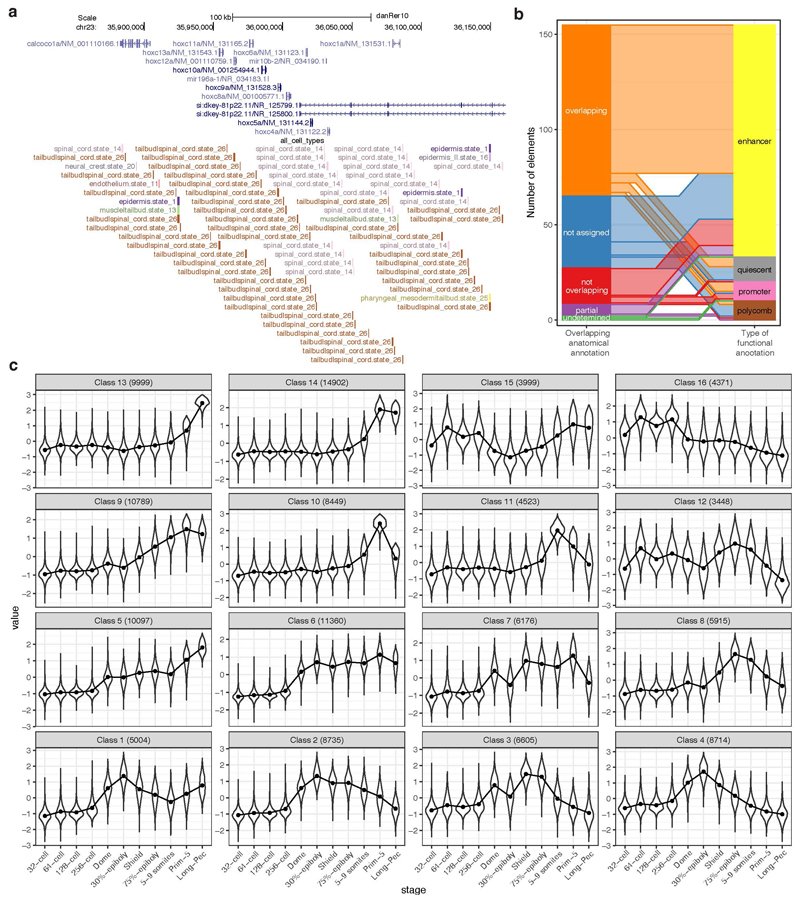
Cell-type and developmental classification of PADREs Cell-type specificity assignment and developmental dynamics of PADREs **a,** An example genomic region shows cell-type assignment of PADREs derived from single-cell ATAC-seq data (bottom track) and the gene model (top track). The name of PADREs contains their cell-type assignment. PADREs in this track are colour-coded by their cell-type assignment as well, each colour representing a different cell-type. The state number in the name corresponds to those defined in McGarvey *et al.*, 2022. **b,** Overlap of matches between the cell-type assignment and activity tissue determined by transgenic assay. Of 155 transgenically validated enhancers active at the Prim-5 stage, 117 have a cell-type specificity assignment. For details of anatomical terms and statistics see Supplementary Table 11. In 72 (62%) assigned transgenic enhancers the scATAC-seq derived anatomical annotation matches at least one of the activity domains of the transgenic reporter (left-hand side of the bar chart). Partial overlap indicates transgene activity in a related tissue, but without no identifiable direct overlap with that of the cell type assignment. Not assigned elements were not registered for cell type specificity by McGarvey *et al.*, 2022. Undetermined elements were not possible to directly compare due to ambiguity of anatomical terms. The functional annotation of transgenically validated PADREs (right column) shows that most transgenic elements have an enhancer relevant ChromHMM registration at the Prim-5 stage. Waterfall plot between the left and right columns indicate overlap between cell type assignment and cis regulatory element category. **c,** Openness of distal (non-promoter) cPADREs throughout development at stages indicated on the x-axis in the defined SOM classes. Numbers in brackets indicate the number of elements in each class.

To assign cell-type specificity to PADREs we integrated them with Prim-5 single-cell ATAC-seq^30^ data (Extended Data Fig. 7a). The majority of anatomical annotation overlapped with transgenically confirmed enhancers and PADRE functional annotation (Extended Data Fig. 7b, Supplementary Table 11). UMAP (Fig. 3i, right) revealed remarkable differences between cell types both within the same tissue and across tissues. PADREs active in neural precursors of the developing central nervous system (CNS) showed a 3-fold increase of H3K27ac compared to those active in differentiating neurons, confirming previous observations about heterogeneity of cell-type population and chromatin dynamics in developing CNS^50,51^. In contrast, PADREs active in muscle cells carried levels of H3K27ac and H3K4me1 comparable to neural precursors, but distinct accessibility profiles (Fig. 3i, bottom).

To understand the temporal dynamics of PADREs, we created a set of consensus PADREs (cPADREs), containing ~140k regions open in at least two neighboring stages (Supplementary Fig. 3a). We clustered non-promoter cPADREs by chromatin accessibility into self-organizing maps (SOMs) (Extended Data Fig. 7c). Fig. 3j (top) shows UMAP locations of 3 out of 16 SOM clusters, which demonstrate remarkable developmental chromatin changes, containing cPADREs active early and subsequently decommissioned (Class 4), active from ZGA onwards (Class 6), and late elements (Class 14). Their chromatin profiles around ATAC-seq peaks were different, with only the early elements depleted of H3K27ac at their peak (Fig. 3j, bottom). With distinct chromatin and conservation profiles, early and late elements represent two separate classes of enhancers.

Finally, we explored the dynamics of PADREs without observable chromatin marks at any stage of development. 2,109 such regions were constitutively open throughout development (Supplementary Fig. 5a), which we termed Constitutive Orphan Predicted Elements (COPEs). They co-localized with constitutive SOM class 6 and 40% of them contained a CTCF motif (Fig. 3k, top; Fig. 3e). In contrast, another non-marked open chromatin set (11,044; termed Dynamic Orphan Predicted Elements; DOPES) was open only in specific developmental stages (Fig. 3k, Supplementary Fig. 5b). They were depleted of promoters, with only 65 (0.6%) overlapping CAGE promoters (Supplementary Table 12). Using data from^24^, we found that 2,513 DOPEs contained active chromatin marks later in adult tissues, but were open to the same extent as active enhancers already in the embryo (Supplementary Fig. 5c). As we are unaware of epigenetically “orphaned” accessible elements in the development, whose chromatin opening precedes or is uncoupled from enhancer-associated histone mark deposition, this may represent a discovery of previously unknown subtype of primed enhancers.

### Developmental specialization of Pol II gene promoters

**Figure 4:**
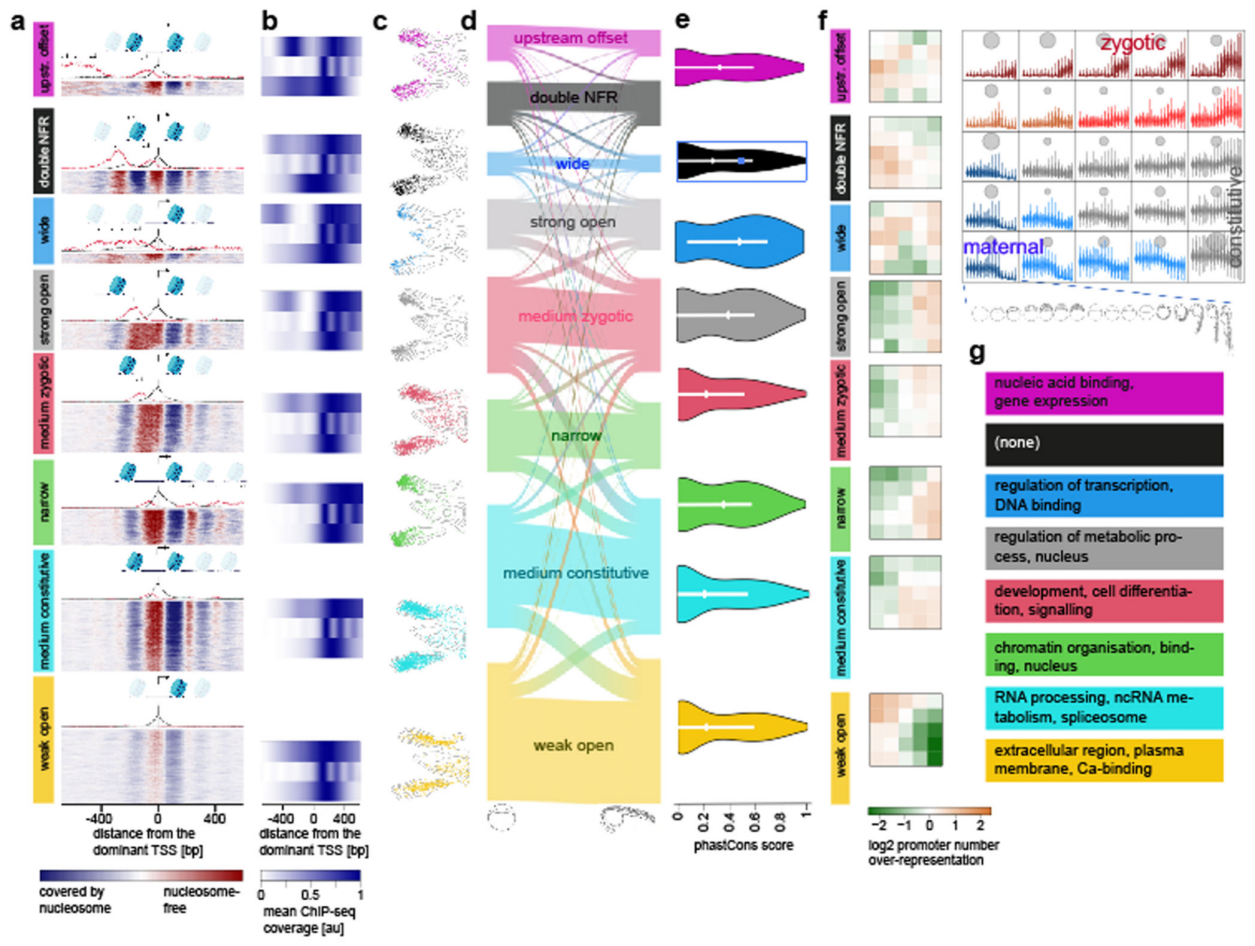
Chromatin architecture classification and developmental specialization of Pol II gene promoters **a,** Heatmap of chromatin accessibility profiles aligned to dominant TSS per promoter at the Prim-5 stage. Nucleosome-free regions (red) are superimposed with nucleosome positioning (blue). Stack height reflects number of promoters. Above each heatmap, combined histograms of CAGE expression are shown. Black, forward TSSs, red, reverse orientation TSSs (The scale is amplified 10× in relation to forward transcription). Nucleosome positioning is symbolized above alignments and black arrows indicate transcription direction; size indicates relative strength. Promoter configuration classes are color-coded consistently in all panels including Supplementary Fig. 6b, Aggregated H3K4me1, H3K4me3 (MNase-digested), H3K27ac ChIP-seq signals for classes as in **a** arealigned to dominant TSS. **c,** UMAP profiles of promoter classes at the Prim-5 stage. UMAPs are cropped to highlight promoter PADREs. **d,** Flow diagram indicates the relationship between promoter configuration class at the Dome stage (left edge, Supplementary Fig. 6) and the Prim-5 stage (right edge). Band width represents the number of promoters. **e,** Violin plot of phastCons vertebrate conservation distribution of promoters. Each class is aligned to **a. f,** Classification of promoter expression during development with self-organizing maps (SOM). On the top right, 5 × 5 diagrams contain violin plots with stage-by-stage expression levels. Blue to red spectrum indicates maternal to zygotic expression dynamics of promoter clusters. Surface areas of grey circles indicate the number of promoters per cluster. Stages of development are symbolized below the SOM array. On the left, mustard: positive and green: negative color spectrum in SOMs indicates the enrichment in promoter overlap between promoter expression classes (SOM) in each chromatin architecture class **a. g,** Enriched GO categories for each promoter architecture class.

To reveal any developmental promoter regulatory principles, we exploited the PADRE chromatin features to functionally classify CAGE-seq defined active RNA Pol II promoters. First, we characterized these promoters at Dome and Prim-5 stages based on their chromatin accessibility at nucleosome-resolution, revealing 8 clusters (Fig. 4a, Supplementary Fig. 6a, Supplementary Table 13). We detected similar clusters in human embryonic stem cells (Supplementary Fig. 6b) indicating conservation of promoter chromatin architecture classes. The classes differed mostly in their upstream configuration, including the width of the nucleosome-free region (NFR), the signal strength of the central NFR, and presence of upstream open regions (Fig. 4a), which followed GC content (Supplementary Fig. 6c). The NFRs only differed in their amplitude between *medium constitutive* and *weak open* (Supplementary Fig. 6a) the latter either reflecting reduced promoter activity or promoters active only in a subset of cells. The NFR variations were characterized by histone mark presence and pattern of upstream opposite strand transcription (e.g., *upstream offset*) with distinct distances between the main TSS and flanking nucleosomes (e.g. *wide* and *strong open*) and TSS profiles (Supplementary Fig. 6d). These classes showed notable differences in histone modification patterns (Fig. 4b), confirmed by the differing UMAP positions of promoter PADREs (Fig. 4c). Apart from *weak open,* each class produces antisense transcription (PROMPTs)^52–54^, including *double NFR*, *wide* and *upstream offset* classes, which showed CAGE expression from both the main NFR and another upstream region, with sense transcription being stronger than antisense (Fig. 4a). Notably, the architecture classes remain stable over developmental time (Fig. 4d, Supplementary Fig. 6e), suggesting they represent distinct regulation mechanisms acting on the genes rather than stage-dependent promoter activity states. *Wide* and *strong open* classes contained the most conserved promoters (Fig. 4e, Supplementary Fig. 6f), and were enriched in transcription regulator genes (Fig. 4g, Supplementary Fig. 6g). However, promoter classes showed distinct dynamic temporal expression (Fig. 4f) with notable enrichment of the *double NFR* class for maternally expressed genes in contrast to the predominantly early and late zygotic *weak open* and *medium zygotic* classes, respectively. The promoter classes also showed distinct gene ontology (GO) enrichment categories (Fig. 4g). Overall, our approach offers a promoter architecture classification for zebrafish and indicates functional specialization and vertebrate conservation of promoter classes.

### Developmental dynamics and locus organization of enhancers

**Figure 5:**
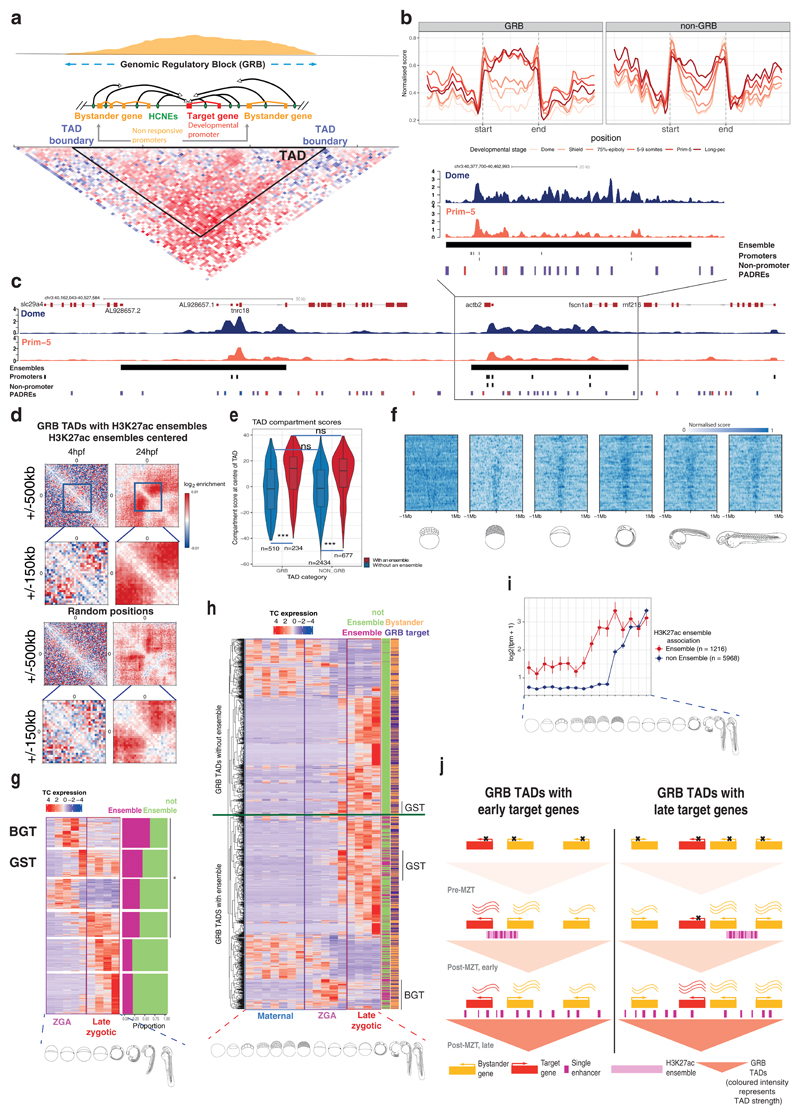
Dynamics and function of open chromatin and H3K27ac topology organization on early embryo development. **a.** Schematic representation of genomic regulatory blocks (GRBs). Middle: basic components of a GRB. GRB Enhancers (green) regulating the target genes span the entire length of the GRB. Top: typical density pattern of conserved non-coding elements in a GRB, most of which overlap enhancers. Bottom: Hi-C contact matrix within a GRB. **b,** Chromatin opening profiles through developmental stages along TADs. **c,** Genome browser view of a GRB TAD showing H3K27ac signals in the Dome and the Prim-5 stages, H3K27ac ensembles (black bar), CAGE promoters (black blocs), and non-promoter PADREs (blue active in the Dome stage, red active in the Prim-5 stage, and purple PADREs active in both stages). Top: left: A zoomed-in genome browser view of an H3K27ac ensemble. **d,** Aggregate contact enrichment centered on ensembles at stages as indicated. e,TAD compartment score distribution. Positive scores represent A-compartments, while negative represent B-compartments. The comparison was done using two-sided two-sample unpaired Wilcoxon test. **g,** Heatmaps of H3K27ac signal across GRB TADs containing ensembles through developmental stages. TADs are ordered by their width in descending order and fixed on the TAD center. h,CAGEexpression patterns of selected gene classes separated by SOM, with the highest and lowest ratios in ensemble-associated genes. Bar plot on the right show the proportion of ensemble-associated genes in each class. BGT and GST classes are marked on the heatmap **i,** Geneexpression pattern of GRB target and bystander genes. The left sidebar shows an ensemble association for each gene. The right sidebar shows the target or bystander assignment for each gene. Genes in TADs with and without ensembles are separated by a green line. BST and GST classes are indicated on the side. **j,** Graph showing mean expression and standard error of GRB target genes associated and not associated with early H3K27ac ensembles. **k,** A model describing the influence of an H3K27ac ensemble on expression of GRB target genes. If the H3K27ac ensemble is in contact with the target gene, it can be expressed early on.

Key genes regulating development are controlled by numerous long-range enhancers, which often overlap with highly Conserved Noncoding Elements (HCNEs) within genomic regulatory blocks (GRBs)^15^ that also often contain other, “bystander” genes that do not respond to those enhancers. The extent of GRBs coincides with those of topologically associating domains (TADs) around developmental genes^55^ (Fig. 5a). We exploited DANIO-CODE annotations to characterize chromatin opening and interaction topology in those poorly understood loci, and their regulatory role in TADs.

**Extended Data Fig. 8:**
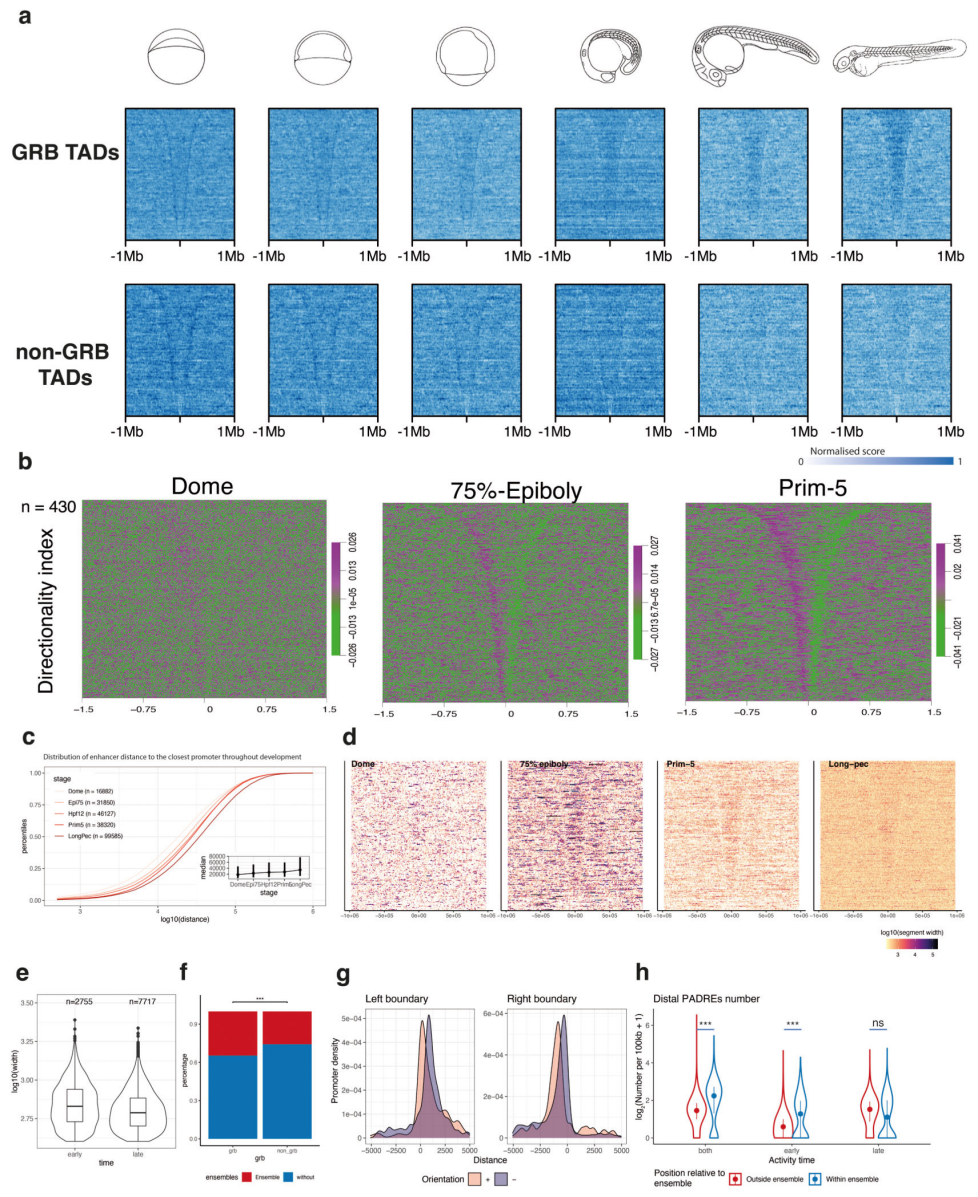
Developmental dynamics of topologically associated domains and H3K27ac ensemble definition **a,** ATAC-seq signals in GRB (top) and non-GRB (bottom) TADs throughout development. TADs are ordered in a descending order from the top of the heatmap. **b,** Directionality index in GRB TADs throughout development. **c,** Distance distribution of enhancer-associated PADREs to the closest promoter within GRB TADs. Bars represents inter-quartile range. **d,** Enhancer-associated ChromHMM segments in GRB TADs throughout development. TADs are ordered in a descending order from the top of the heatmap. Segments are coloured based on the logarithm of their length. Early stages are dominated by fewer large blocks, which start to be enriched within TADs only at 75%-Epiboly. In late stages, short segments are distributed uniformly throughout the entire TAD length. **e,** Width distribution of concatenated enhancer-associated ChromHMM segments. Singletons shorter than two bins (400 bp) were excluded. The number of segments is shown above each violin plot. **f,** Ratio of GRB and non-GRB tads containing H3K27ac ensembles. **g,** the density of CAGE promoters on ensemble boundaries. **h,** the number of non-promoter PADREs per 100kb in TADs containing ensembles. The x-axis shows the developmental stage in which the PADRE is H3K27ac marked (early, late, or both). The location of promoters in respect to the ensemble is shown in different colours. The numbers were compared using two-sided two-sample unpaired Wilcoxon test.

We distinguished *GRB TADs,* characterized by a high density of extreme non-coding conservation, from *non-GRB TADs*. In the regions corresponding to late (Long-pec) embryo TADs, chromatin started opening at the boundaries as early as the Dome stage and remains open thereafter (Fig. 5b, Extended Data Fig. 8a). GRB TADs showed a strong increase in accessibility across the entire TAD, whereas in non-GRB TADs the increase was mild and occurred later (Fig. 5b). TADs started to form early but formed fully only at later developmental stages^56,57^ (Extended Data Fig. 8b). We found more promoter-proximal enhancers in early, and distal enhancers in late stages, (Extended Data Fig. 8c) in line with similar findings by contact analysis^56^.

When we estimated the activity of enhancer candidates by H3K27ac in TADs, we observed that such elements in late stages are numerous, short, and distributed throughout the entire TAD length. In contrast, many fewer PADREs were active early at Dome stage, and they often occurred in clusters with uninterrupted H3K27ac signal connecting them (Extended Data Fig. 8d,e; Fig. 5c). We detected ~1,600 such clusters^58^, of which ~1,300 fell in TADs and were enriched in GRB TADs (Extended Data Fig. 8f). These clusters were reminiscent of super-enhancers^58,59^, although more numerous than 231 reported in mouse^58^ and 411 in zebrafish^57^. Given their unusual scale and early appearance before lineage determination (when previously reported super-enhancers appear), we distinguished them from super-enhancers and called them *H3K27ac ensembles*. We hypothesized that they might be associated with the lack of fully formed TADs in the early stages when enhancers are used proximally to early active promoters. To test this, we investigated the relationship between the chromatin interactions and activity of H3K27ac-ensemble-associated genes during early vs. late embryogenesis.

We found that promoters were enriched at the boundaries of H3K27ac ensembles (Extended Data Fig. 8g) and that the ensembles contain most candidate enhancer PADREs detected in early stages (Extended Data Fig. 8h). In contrast, the PADREs active only later in development represented long-range enhancers, distributed across the entire TAD (Extended Data Fig. 8d), and not enriched in ensembles (Extended Data Fig. 8h). Moreover, H3K27ac present along the entire length of the ensemble became restricted to individual peaks associated with PADREs by Prim-5 (Fig. 5c, zoomed panel).

**Extended Data Fig. 9:**
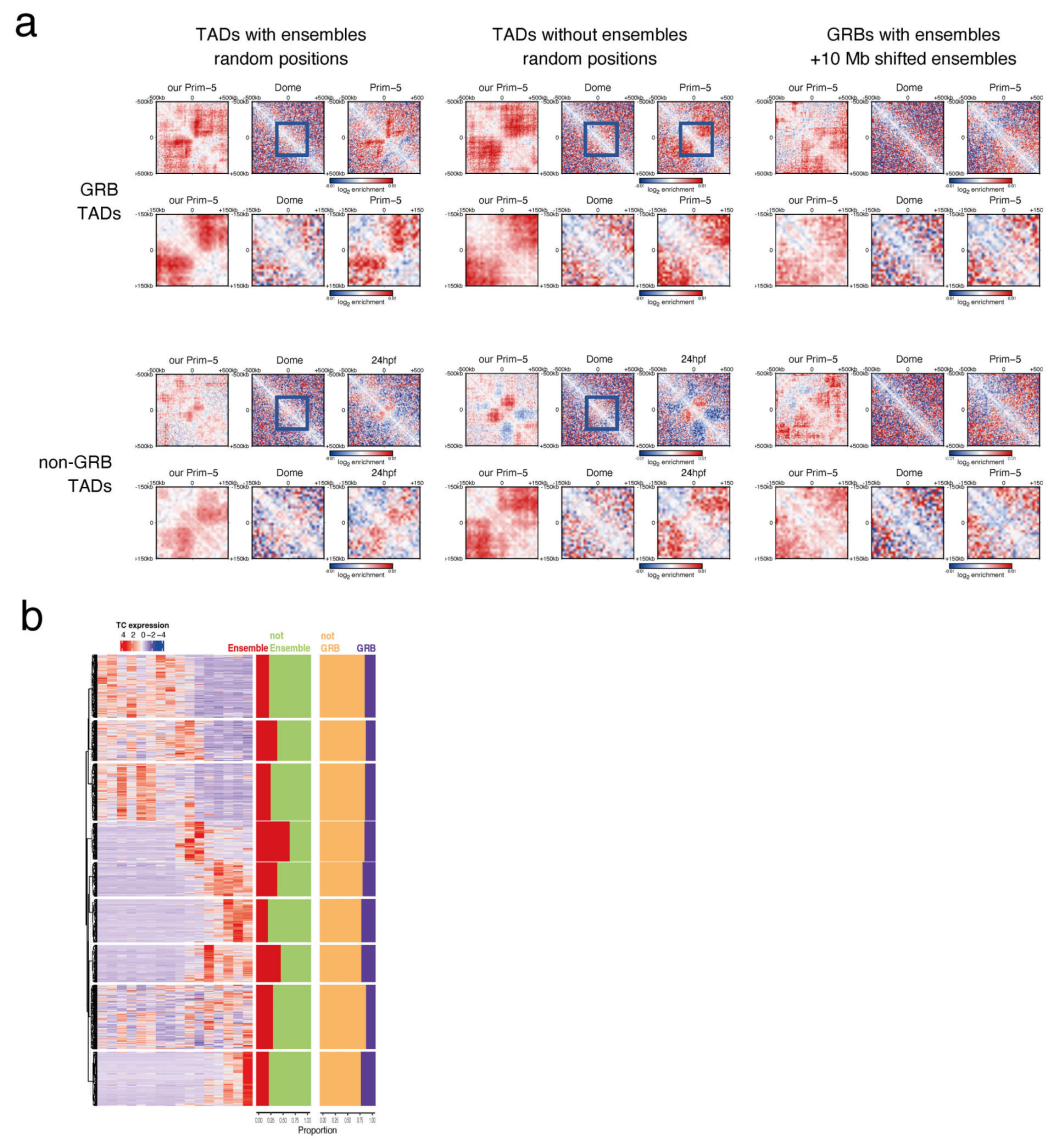
H3K27ac ensemble contact enrichment and CAGE expression patterns of gene classes separated by SOM **a,** Controls for contact enrichment around H3K27ac ensembles. All regions were downsampled to n=56 to match the number of 50 kb - 150 kb size ensembles. Labels are as in Figure 5g. The controls included random positions within the same TAD **(a),** random positions within TADs without ensembles, and 10MB shifted positions, for GRB TADs (top row) and non-GRB TADs (bottom row). The controls include published data for the Prim-5 stage, as well as new, unpublished data with higher resolution (Prim 5).. **b,** CAGE expression patterns of gene classes separated by SOM. Bar plots in the middle show the proportion of ensemble-associated and GRB genes in each class respectively

Consistent with an H3K27ac ensemble role in early gene regulation, we observed increased Hi-C contacts within them at Dome in both GRB and non-GRB TADs. By Prim-5, strong contacts spread throughout the entire TAD (Fig. 5d, Extended Data Fig. 9a). TADs with H3K27ac ensembles present at Dome belonged to the active A-compartment at Prim-5 (Fig. 5e), arguing for a role for H3K27ac ensembles in the timely opening of chromatin in their host TADs. Indeed, in GRB TADs, the H3K27ac mark propagated from H3K27ac ensembles to fill the entire TAD in later stages (Fig. 5f).

To examine how H3K27ac ensembles influence gene expression, we classified promoters within TADs by expression dynamics using SOM (Extended Data Fig. 9b). H3K27ac-ensemble-associated promoters mostly sequestered into clusters with the highest expression in early post-ZGA stages (Fig. 5g). We termed the top two H3K27ac-ensemble-associated classes Blastula-Gastrula Transition (BGT) and Gastrula-Segmentation Transition (GST), based on the peak expression time. The two major gene classes in GRB TADs were ubiquitously expressed (GRB bystanders), and late zygotic expressed (likely GRB target genes). However, in GRB TADs with an ensemble, we observed a BGT gene class, not present in GRB TADs without an ensemble, as well as more genes in the GST class. Both classes were enriched in ensemble-associated genes (Fig. 5h). Moreover, there was a clear trend of earlier expression in H3K27ac-ensemble-associated GRB target genes, compared to other GRB target genes (Fig. 5i), suggesting that ensembles participated in the activation of early acting developmental genes, including those later dependent on long-range regulation. Moreover, if the target gene is not in contact with the H3K27ac ensemble, it can only become expressed once long-range interactions are present (Fig. 5j).

### Functional conservation of epigenetic subdomains

**Extended Data Fig. 10:**
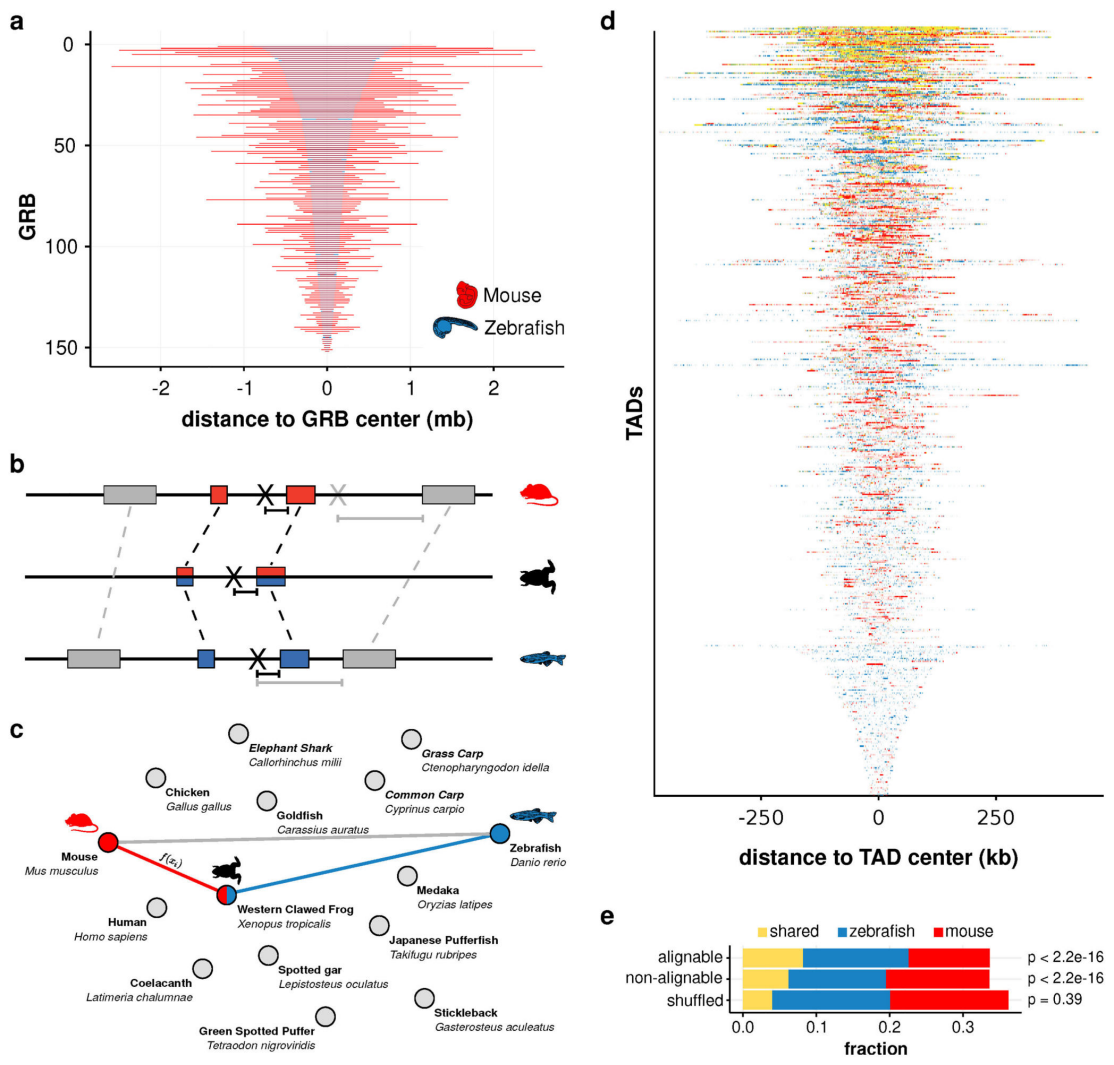
Epigenetic domains comparison between zebrafish and mouse **a,** Comparison of sizes of genomic sequences covering orthologous GRB-containing TADs. TADs are ranked by size, largest on top. **b,** Schematic illustration of the projection of an example genomic location X between zebrafish and mouse by interpolation using the direct alignments (grey rectangles) and the alignments via a bridging species (blue and red rectangles, Xenopus in this example). projections are indicated as a black X in the respective species). Dashed lines connect pairwise sequence alignments. The projected locations of X in mouse are indicated in grey (direct alignments) and black (via bridging species). **c,** Example graph comprising 15 species (nodes). For any genomic location, the shortest path through the species graph yields the combination of species which maximizes projection accuracy. **d,** H3K27me3 overlap profiles of all GRB TADs. TADs are ordered by their relative amount of shared signal. Bins are in the original genomic order. **e,** Fractions of bins with shared or species-specific H3K27me3 enrichment. Bins are classified as alignable (n=22,403) if they overlap a direct sequence alignment between zebrafish and mouse and as non-alignable otherwise (n=97,767). P-values are obtained by Fisher’s exact test.

**Figure 6:**
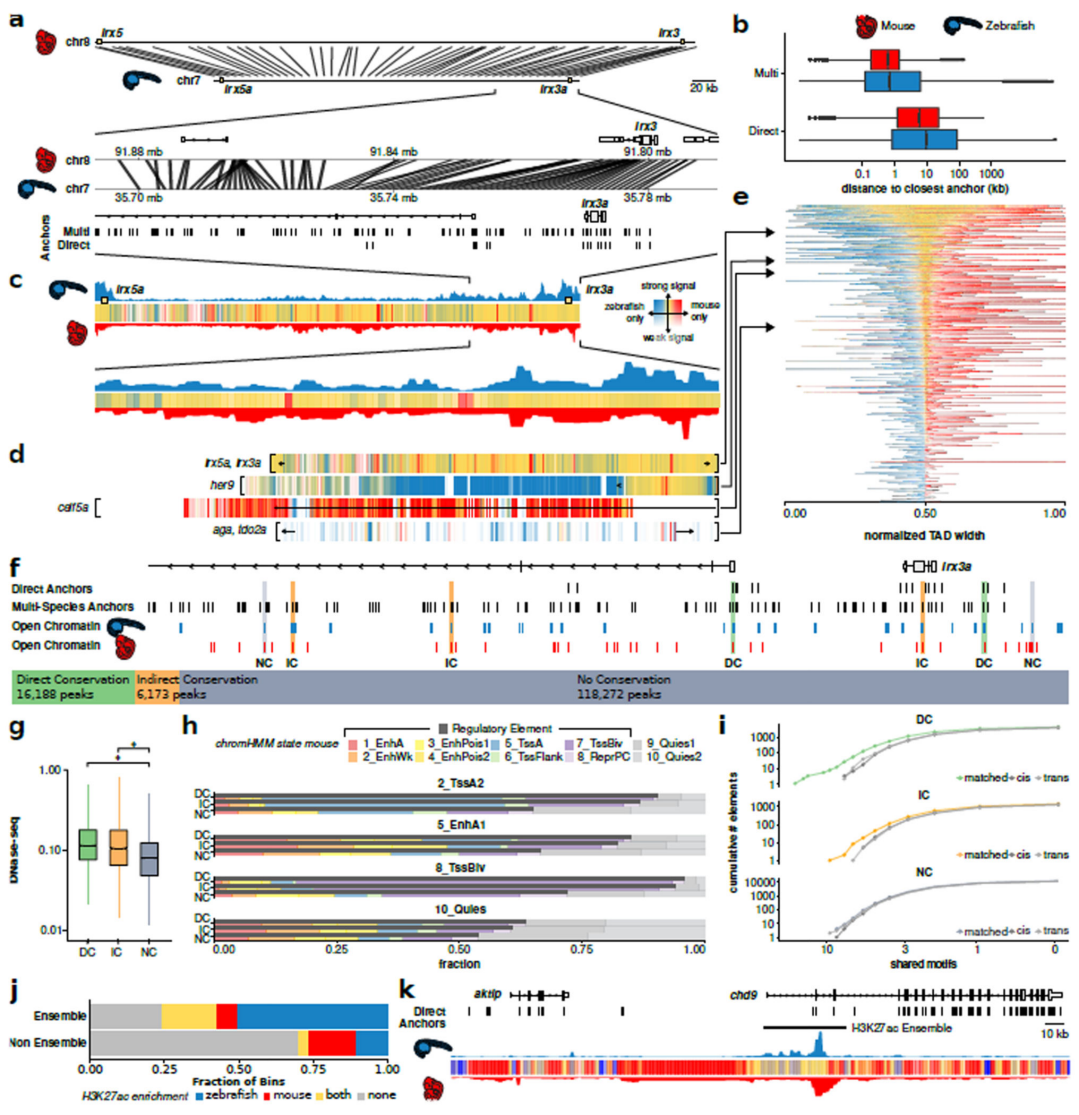
Synteny projections revealconservation of epigenetic features between zebrafish and mouse **a,** Cross-species comparison of the *irx3/5*(a) TAD between zebrafish and mouse and a zoom-in on the locus around *irx3*(a). Connecting lines represent projections of bin centers from zebrafish to mouse. **b,** Distribution of distances from the bin centers (n = 528,830) to their closest anchors in zebrafish (blue), and from their projections to their closest anchors in mouse (red), using the direct and the multi-species projection approach. **c,** Epigenetic comparison of the *irx3*/5(a) TAD. H3K27me3 overlap in mapped regions is indicated as colored bars (yellow - mutually enriched, blue - zebrafish specific, red - mouse-specific, see Methods). Opacity reflects signal amplitude and is proportional to the maximum H3K27me3 signal in both species. **d,** H3K27me3 overlap profiles for four selected GRB TADs. TAD boundaries are indicated with square brackets. **e,** H3K27me3 overlap profiles of all GRB TADs. TADs are ordered by their relative amount of shared signal. Bins are ordered by the amount of shared signal, bins with shared signal appear in the middle, bins with zebrafish and mouse-specific signals are left and right, respectively. A view of the TADs with their genomic bin order is given in Extended data. Figure 10 d,f. Classification of zebrafish ATAC-seq peaks in the *irx3a* locus into DC, IC and NC based on overlaps with direct anchors, multi-species anchors, and mouse DNase-seq peak projections (see Methods). **g,** Distribution of DNase-seq signal in the mouse genome around the projected regions of the zebrafish ATAC-seq peaks (n = 140,633). Asterisks above the bars indicate the effect size category based on Cohen’s d: very small (not indicated), small (*), medium (**), large (***), very large (****). **h,** Cross-species comparison of ChromHMM functional states. **i,** Cumulative distribution of shared motifs in mouse DNase-seq peaks overlapping zebrafish ATAC-seq peaks. **j,** H3K27ac enrichment (signal ≥ 80th percentile) within (n = 11,083) and outside (n = 93,020) of enhancer ensembles (*P* < 2.2 × 10^-16^, Fisher’s exact test). **k,** Cross-species comparison of H3K27ac profile around an H3K27ac ensemble neighboring the zebrafish *aktip* gene.

Next, we investigated whether our annotation of non-coding elements could be exploited to predict functionally conserved CREs among vertebrates. Existing comparative methods rely on direct alignments between species of interest^60,61^ However, the large evolutionary distance between fish and mammals limits the power of comparison due to loss of non-coding sequence similarity. We developed a method to predict functional conservation across large evolutionary distances and genomic scales independent of direct sequence alignment, exploiting the fact that functional elements often maintain collinear syntenic positions, while their spacing scales with genome size, particularly in GRB TADs^15,55,62,63^ We selected 13 high-quality bridging species reference genomes and using stepped pairwise sequence alignment (Extended Data Fig. 10 and Methods), which allowed us to map coordinates between genomes of varying sizes, identified reference points (multi-species anchors; Fig. 6a) between genomes, enabling identification of syntenic regions through interpolation of relative syntenic positions between anchor points.

We then compared zebrafish and mouse GRB TADs, which differ in size approximately two-fold (Extended Data Fig. 10a). We defined GRB TADs as the 1,000 TADs with the highest CNE density, split them into 1-kb bins, and mapped the bin centers from zebrafish to mouse. Using our multi-species approach over direct alignment reduced distances from the bin centers to their closest anchor by a factor of 16 in zebrafish and 29 in mouse (Fig. 6b).

We asked whether this method could discover conserved epigenomic subdomains by comparing epigenomic feature distribution across genomes. We used H3K27me3 ChIP-seq data from phylotypic stages in zebrafish (Prim-5) and mouse (E10.5; see Methods). H3K27me3 coordinates from zebrafish were projected onto the mouse genome, recovering mouse H3K27me3 features in the corresponding region. An example at the *irx3a* locus (Fig. 6c) shows H3K27me3 enrichment correlates between zebrafish and mouse, even in the absence of direct sequence conservation. On a genome-wide level, H3K27me3 enrichment is significantly more likely to be shared between zebrafish and mouse for both directly alignable and non-alignable genomic regions (Extended Data Fig. 10e), suggesting epigenomic subdomains and functional elements can be conserved in location and span. We see more GRB TADs showing regions of strong similarity in H3K27me3 extent, while others, such as TADs containing *her9* or *celf5a*, show more zebrafish or mouse-specific signal enrichment, and still others show little enrichment (Fig. 6d-e).

We next looked at conservation of functional elements marked by open chromatin. We classified zebrafish ATAC-seq peaks in the GRB TADs as directly conserved (DC) if they fall in a region of direct sequence alignment with mouse (16,188 elements, 11.5 %), indirectly conserved (IC) if they do not directly align (6,137 elements 4.4%) but were alignable through bridging species, and non-conserved (NC) for all other peaks (e.g. *irx3a* in Fig. 6f). Notably, DC and IC elements shared regulatory features with their matched counterparts in mouse, including DNase hypersensitivity and ChromHMM feature classification, compared to NC elements (Fig. 6g, h). DC and IC regions were also more likely to share TF binding site (TFBS) motifs compared to non-overlapping, randomly sampled mouse DNase-seq peaks within and across TAD boundaries (*cis* and *trans* in Fig. 6i; Supplementary Table 14). These results suggest a similar level of functional conservation of DC and IC elements, even though IC elements lack direct alignability. Next, we tested whether the early developmental H3K27ac ensembles detected in zebrafish embryos (Fig. 5) are conserved in mouse using our anchoring-based approach. As shown in Figure 6j,k, H3K27ac signal in mouse was significantly enriched in zebrafish ensembles, suggesting these ensembles are evolutionarily conserved epigenetic subdomains in vertebrates. Genes associated with these conserved ensembles are listed in Supplementary Table 15. Our comparative epigenomic approach has maximized the identification of putative functional elements and epigenetic subdomains conserved between zebrafish and mouse and highlight the utility of the DANIO-CODE annotations for discovery of vertebrate-conserved mechanisms.

## Discussion

Here we describe the establishment and provision of a zebrafish developmental genomics resource as a track hub and downloadable resource within a Data Coordination Center, which is designed for expansion by continued incorporation of future zebrafish genomic data. Our track hub allows visualization of developmental non-coding functional annotations in common genome browsers.

We have annotated over 140,000 candidate developmental CREs including enhancers and promoters. There is a need for the classification of CREs to reflect their distinct temporal and spatial dynamics and modes of functionality. Recognizing this, we improved the classifications of enhancers and promoters with novel subcategories using dimensionality reduction on chromatin accessibility and nucleosome-level histone modifications. CRE subclasses include DOPEs and COPEs, which may carry yet unmapped histone marks^64^ and merit further investigation. We demonstrate distinct local chromatin architecture of CREs in developmental stages and in developing cell types. Moreover, we classified promoters into potentially novel chromatin architecture classes, which we also detected in mammals, and which are distinctly used by subsets of genes. We have integrated our CRE annotations with chromosome topology and explored the dynamic organization of CRE interactions during development. We identified large H3K27ac-marked ensembles, which are distinct from previously described super-enhancers targeting lineage determining genes. We suggest ensembles function in nuclear topology organization at local interaction hubs around early active loci during the initial formation of TADs.

The datasets used in this study are bulk whole-embryo samples, which can mask chromatin state dynamics of rare cell populations or varying cell cycle states. Nevertheless, we were able to identify distinct subclasses of candidate CREs such as promoter classes and COPEs by comparing intersections of independent chromatin features. In this effort, stable chromatin features served as references to compare the varying dynamics of overlapping chromatin states, mitigating bulk averaging artefacts. The expansion of single-cell genomic data by the zebrafish community will help in further stratifying *cis*-regulatory classes. Meanwhile users are encouraged to browse tracks for enhancer marks emanating from small cell numbers that may be masked by thresholding, and to integrate tissue and cell-type specificity information. Such integration will generate further layers of functional annotations, including TF expression and binding sites^65^ and help in identification of gene regulatory network components acting in lineage determination^66^.

The DCC and the functional annotation track hub will thus serve as a foundation for future single-cell studies of transcriptomes^67–69^, open chromatin^30^, and others, as demonstrated with scATAC-seq data here. The functional annotations presented will also aid in targeted manipulation of genomic elements. For example, our high-resolution promoter annotation will aid reagent design for gene regulation assays^70^, transgenic cell labeling^71^, and transcription blocking.

The utility of our functional annotations extends well beyond zebrafish development. We developed an approach that detects functional equivalence of regulatory landscapes in the absence of sequence conservation. Our multi-species anchoring approach facilitated the identification of non-sequence conserved positional equivalents with enrichment for shared epigenetic domains (H3K27me3 and H3K27ac) and syntenic enhancer TFBS content, highlighting the predictive value and functional relevance of epigenetic subdomains within syntenic TADs. This zebrafish resource thus expands on and complements the existing functional genome mapping efforts in mammals and modENCODE species.

## Methods

### Resources and data availability

The resources produced by this publication, along with their location are as following:

1. Overview of the DANIO-CODE consortium and contributors (https://www.birmingham.ac.uk/generic/danio-code/index.aspx)
2. DANIO-CODE Data Coordination Center (DCC) (danio-code.zfin.org)
3. DANIO-CODE track hub for UCSC browser (danRer10: http://genome.ucsc.edu/cgi-bin/hgTracks?db=danRer10&hubUrl=https://danio-code.zfin.org/trackhub/DANIO-CODE.hub.txt danRer11: http://genome.ucsc.edu/cgi-bin/hgTracks?db=danRer11&hubUrl=https://danio-code.zfin.org/trackhub/DANIO-CODE.hub.txt)
4. Session for WashU EpiGenome Browser (https://github.com/DANIO-CODE/DANIO-CODE_Data_analysis/tree/master/Figures/Figure1#figure-1c=)
5. Motif Activity Response Analysis (MARA) (https://ismara.unibas.ch; DANIO-CODE results: https://ismara.unibas.ch/danio-code)
6. Regulatory motifs and regulatory site annotations (https://swissregulon.unibas.ch/sr/downloads)
7. Code repository for DANIO-CODE processing pipelines (gitlab.com/danio-code)
8. Code repository for data analysis in this paper (https://github.com/DANIO-CODE/DANIO-CODE_Data_analysis)
9. Videos with tutorials and example usages of the resource: https://youtube.com/playlist?list=PLiWQCe7dGqm6AtA0oP7qIaEQNa-7Z7fh5

### Animal work

All animal work associated methods are presented in Supplementary Methods. Only early zebrafish embryos up to the free-feeding stage (5 days post fertilization) were used in this study. Zebrafish embryos/larvae up to the free-feeding stage are not considered as protected animals by law in the UK and are not subjected to animal experimentation regulations. Breeding and maintenance of adult zebrafish strains was carried out in a designated facility under Home Office project licenses 40/3681 and P51AB7F76 assigned to the University of Birmingham, UK.

### Data Collection

We started the DANIO-CODE data collection aiming to capture a wide range of developing stages in zebrafish from a broad range of genomic, epigenomic and transcriptomic assays.

Members of the zebrafish community were invited to provide their published as well as unpublished data to the DANIO-CODE consortium. Benefiting from experiences of consolidating data in the decentralized data production of the modENCODE consortium^72^, we developed the DANIO-CODE Data Coordination Center (DCC)^26^ (https://danio-code.zfin.org). The DCC facilitated data collection as well as data annotation and subsequent data distribution.

Demultiplexed FASTQ files were provided by community members to the DCC file system. Using the DCC-web frontend, the community members were guided through an annotation process to annotate the data they provided. The DCC data model is derived from Sequence Read Archive (SRA) data structures^73^ and employs controlled vocabularies based on ZFIN nomenclature^74^. In addition to the community-provided data, DANIO-CODE annotators strategically selected additional published datasets to complement developmental stages or assays so far underrepresented in the DCC. These datasets were annotated by the DANIO-CODE curators based on the respective publications.

Consistent data and annotation formats allowed the consistent processing of all data in the DCC. For this, we developed computational workflows for all the data types and implemented these workflows for the DNAnexus system (https://www.dnanexus.com). Data and annotation quality control measures were established for all data in the DCC.

As a result, all datasets present in the DCC are described in terms of the overall study design, biosamples, library preparation methods, sequencing details as well as in data processing and quality control aspects. Snapshots of the DCC are kept as data freezes to facilitate the handling of newly added data. An interactive data and annotation view and export is provided at https://danio-code.zfin.org/dataExport.

### Transcripts identification

Wild-type embryonic paired-end and stranded RNA-seq samples (DCD000141SR, DCD000225SR, DCD000247SR, DCD000433SR, DCD000426SR, DCD000324SR)^31,33,75–78^ were selected from a total of 528 DANIO-CODE RNA-seq and aligned to GRCz10 using STAR aligner v. 2.5.1b^79^. StringTie v.1.33b^80^ was used to call transcripts, which were then all assembled using TACO^81^, generating a total of 194,508 transcripts in canonical chromosomes. Transcript quantification was done using Salmon v.0.11.2^82^. We removed read through, mono-exonic and transcripts overlapping 3 or more Ensembl genes. All protein coding transcripts above 200 kb and long non-coding RNAs above 100 kb were excluded (permissive set). We defined transcripts that are expressed in minimum 2 closest stages as the robust set. To get high confidence transcripts, we added those which have consensus CAGE TCs in the proximity (± 500 bp), yielding 70,354 transcripts (permissive set) and 55,596 transcripts (robust set). Out of 35,117 Ensembl genes (v.91), 22,065 and 23,568 genes were covered in our robust and permissive sets, respectively.

### Promoterome construction

First, all reads mapping to poorly assembled genomic regions or otherwise blacklisted^83^ regions were excluded from the set of cTSSs. After an initial application of CAGEr^84^ we discovered systematic differences between nAnTi and tagging CAGE samples both at the number of Transcription Start Site Clusters (TCs) number and summed promoter/gene expression across samples produced with the two CAGE protocols. Particularly, the fraction of CAGE signal coming from annotated exons was elevated in nAnTi samples skewing the statistics. To a varying extent this phenomenon (known as exon painting/carpeting) has been previously observed and attributed to recapping of degraded mRNAs. Since the majority of true TSS are initiated at either YC or YR dinucleotides^9^, we analyzed dinucleotide frequencies at initiation sites and confirmed an increased proportion of other (non-YC and non-YR) dinucleotides in nAnTi compared to tagging samples. We therefore decided to remove all CAGE tags not initiated at YC or YR dinucleotides.

The remaining set was of TSSs was power-law normalized^85^ to a common exponent alpha = 1.1 and 5 to 1,000 tags fit range, and the TCs were produced using the following parameters of CAGEr: threshold = 0.7, thresholdIsTpm = TRUE, nrPassThreshold = 1, method = “distclu”, maxDist = 20, removeSingletons = TRUE, keepSingletonsAbove = 5. This yielded a comparable number of TCs across samples without an obvious bias towards high numbers in nAnTi samples. The number of TCs moderately increased in post-ZGA samples.

To compare expression levels across samples we called consensus clusters (genomic regions not assigned to any particular sample unlike TCs) with settings tpmThreshold = 1.0, qLow = NULL, qUp = NULL, maxDist = 20. To further filter weak or spurious tag clusters we kept consensus clusters which were expressed in at least 2 consecutive developmental stages. Specifically, we required that there exist a TCs within a consensus cluster in both consecutive stages with at least 1.0 tpm expression. This yielded 27,781 consensus clusters. We calculated expression of each consensus cluster by summing all YC and YR-initiated tag CAGE tags from within the cluster across stages. This approach differs from CAGEr implementation which includes expression only from TCs within a consensus cluster and is subject to generating noise at lowly expressed regions due to the tpmThreshold parameter.

To visualize obtained expression levels we made a 2D PCA plot, which correctly grouped nAnTi CAGE and tagging CAGE samples from the same stage.

### Annotation of alternative transcript and alternative promoter

A gene can have multiple transcripts/isoforms that differ in their transcription start sites (TSSs) by few nucleotides to tens of kilobases. When a gene has multiple transcripts, Ensembl assigns the longest transcript as a reference transcript and its promoter as a reference promoter. To comprehensively assign CAGE peaks to transcripts, we analyzed transcripts models from Ensembl, RefSeq, and novel RNA-seq transcripts from DANIO-CODE. Thus, we focused only on the transcripts that are supported by CAGE peaks. Similar to the Ensembl method, we annotated the longest transcript as the reference transcript and its promoter as the reference promoter. Remaining transcripts whose TSSs were proximal (<300 nucleotides) to the assigned reference transcripts were excluded. On the remaining distal transcripts, the longest transcript was assigned as an alternative transcript and excluded other transcripts with proximal (<300 nucleotides) transcription start sites. Some genes have more than one alternative transcript, thus we iterated this process to annotate additional alternative promoters that are distal from assigned reference or alternative transcripts.

To annotate alternative promoters utilized during mouse embryonic developmental stages, we analyzed FANTOM5 CAGE-seq data^28^ from four embryonic stages (E11 days, E12 days, E13 days and E14 days), which are similar to the zebrafish stages analyzed. We analyzed Ensembl, RefSeq and RNA-seq transcripts models downloaded from UCSC table browser. We used similar method as described above to annotate alternative promoters in mouse. To identify orthologs of alternative transcripts/promoters, we downloaded mouse/zebrafish ortholog table from Ensembl^86^.

### Motif activity analysis

In order to curate a set of regulatory motifs for zebrafish we first collected all Pfam models that correspond to DNA binding domains (DBDs). To define a set of zebrafish transcription factors (TFs) we extracted a representative protein sequence for each zebrafish gene, ran HMMER with these Pfam models, and extracted the DBD sequences for each protein with significant hits. Starting from a previously curated collection of regulatory motifs for human and mouse^37^, we extracted the DBD sequences of the human and mouse TFs associated with these regulatory motifs. Using BLAT^87^, we then aligned all zebrafish DBD sequences to the human/mouse DBD sequences and associated zebrafish TFs with the human or mouse TF (and regulatory motif) that best matched their DBD sequences. Note that multiple zebrafish TFs can thus end up being associated with the same regulatory motif. These procedures led to 814 zebrafish TF genes being assigned to 581 unique regulatory motifs.

For each promoter, we defined the proximal promoter region as the region from 500 base pairs upstream to 500 base pairs downstream of the CAGE TSR. For each proximal promoter region, we obtained the orthologous regions from the goldfish, common carp, and grass carp genomes using LAST^88^ and multiply aligned the orthologous regions using T-coffee^89^. We also obtained a phylogenetic tree of the four species from the observed fractions of conserved nucleotides in the promoter alignments of each pair of species. For each regulatory motif, we then ran MotEvo^90^ on these multiple alignments to obtain transcription factor binding site (TFBS) predictions genome-wide. Using these TFBS predictions, we constructed a sitecount matrix *N* for the MARA analysis, where each component *N_pm_* corresponds to the sum of the posterior probabilities of all binding sites for motif *m* in promoter *p*. Motifs whose site counts across promoters genome-wide had a higher Pearson correlation than *r* = 0.6 were grouped into motif groups, leading to 489 motif groups. ISMARA analysis was then performed on the CAGE expression data across the developmental time course^90^.

### Functional segmentation of the genome

We identified *cis*-regulatory elements genome-wide using their characteristic ChIP-seq signal. For example, acetylation of lysine residue 27 and monomethylation of lysine residue 4 on the histone H3 (H3K27ac and H3K4me1) are features of active chromatin. The modification H3K4me3 is characteristically found on promoters, while H3K27me3 represents Polycomb repressed chromatin. Those modifications were localized ChIP-seq. We used ChromHMM^44,45^ to segment the genome into regions containing specific chromatin marks. We captured the epigenetic state of the genome in 5 different development stages. We used published data for the Dome, 75%-epiboly, 5-9 somites, Prim-5, and Long-pec stages^4,31,91–94^, as well as newly produced data for the 5-9 somites and Long-pec stages. After optimization, we found ten optimal latent states based on the emission parameters of chromatin marks. The states were matched between stages and manually assigned a function using The Roadmap Epigenome Project^21^ annotation as a reference. The identified functional elements were annotated as followed:

1. Active TSS 1 (1_TssA1),
2. Active TSS 2 (2_TssA2),
3. TSS Flanking region 1 (3_TssFlank1),
4. TSS Flanking region 2 (4_TssFlank2),
5. Active enhancer 1 (5_EnhA1),
6. Enhancers flanking region (6_EnhFlank),
7. Primed enhancer (7_EnhWk1),
8. Poised elements (8_Poised),
9. Polycomb repressed regions (9_PcRep),
10. Quiescent state (10_Quies)

The active promoters and promoter flanking regions, in addition to active chromatin marks, show strong emission of H3K4me3. Moreover, the promoter-associated states are mostly found on and around the annotated TSS. The states missing H3K4me3 and not found around TSS were annotated as enhancer-related. Depending on if both H3K27ac and H3K4me1 were present, as well as the strength of the emission, the enhancer states were divided into active enhancers (strong H3K27ac and H3K4me1 emission, but no H3K4me3), enhancers flanking (weak H3K27ac emission, mostly found around active enhancers), and primed enhancers (H3K4me1 emission only). States emitting H3K27me3 were annotated as Polycomb-related. In addition to H3K27me3, when active marks were present, the state was assigned as poised; otherwise, it was assigned as Polycomb repressed. When no marks were present, the region was assigned as quiescent. Most of the genome shows no marks at all.

### Predicted ATAC-supported Developmental Elements (PADREs)

We constrained our subsequent analyses to the regions in the genomes which are open, i.e., depleted in nucleosomes as identified by ATAC-seq. We identified stage-specific open chromatin regions consistent between replicates with the irreproducibility discovery rate^95^ less than 0.1 in seven developmental stages (4 pre-ZGA stages, newly produced datasets, and 7 post-ZGA stages of which 30%-epiboly is newly produced and the other samples were published previously^78^). We termed those regions as predicted ATAC-supported developmental regulatory elements (PADREs). The reason for terming them in this way was to distinguish them from ENCODE cCREs in two segments: 1) they contain open regions even without the support of functional marks, and 2) we wanted to emphasize the developmental aspect of the defined set of elements. All stage-specific PADREs were merged to form a set of regions called consensus PADREs (cPADREs). Two different sets of cPADREs were defined. The permissive cPADREs comprise all PADREs merged and number around ~240k elements. The strict set considers regions that are open in at least two neighboring stages. This set counts ~140k elements. All cPADRE analyses in this paper were done on strict cPADREs. We assigned each ATAC-seq peak a functional annotation based on overlaps with the ChromHMM state in available stages.

### UMAP visualization

We developed a method that considers various signals around the open chromatin summit comprehensively. In brief, we constructed a feature matrix using ATAC-seq, H3K4me3, H3K27ac, H3K4me1 ChIP-seq tags, as well as nucleosome position calculated by NucleoATAC^96^ (Extended Data Fig. 6a-c). Nucleosome signal was included because some factors have well-positioned nucleosomes around their binding sites and could separate those factors from others. In brief, the peak summit is extended for 750 bp in each direction and split into 13 bins (R1 - R13). For each bin, the number of tags for the aforementioned assay types is counted, and the mean nucleosome signal in each bin was calculated using the genomation package. It resulted in five score matrices, each having the number of rows the same as the number of open chromatin regions and 13 columns (one for each bin around the peak summit). Those matrices were standardized by scaling the values and centering the mean to 0. The standardized matrices were concatenated column-wise, giving a total of 65 columns. Using the UMAP algorithm^97^, the number of features was reduced from 65 to 2, making it possible to plot each open chromatin region into a 2D plot.

For the conservation analyses, the cyprinid (grass carp, common carp, goldfish, and zebrafish) phastCons score from Chen *et al.*^98^ were used.

### Constitutive Orphan Predicted Elements (COPEs) and Dynamic Orphan Predicted Elements (DOPEs)

Constitutive elements were defined as the intersection of distal PADREs at every developmental stage. COPEs were defined as constitutive, annotated as quiescent at every developmental stage. DOPEs were defined as cPADREs, annotated as quiescent at every developmental stage. DOPEs were further classified as adults-marked DOPEs if they overlapped H3K27ac marked regions in any of the adult tissues^24^.

### Promoter classification by open chromatin

For each Transcription Start Region we defined a reference point as the TSS with highest mean post-MBT expression “dominant TSS” (tpm values of samples ranging from the Shield to Long-pec stages) and required that it amounts to at least 0.2 tpm. This further reduced the set of consensus clusters to 21,914 elements. We then merged ATAC samples from the Prim-5 stage and extracted Tn5 cut sites from both ends of ATAC reads while correcting for Tn5 overhang, smoothed with a Gaussian kernel with standard deviation 3bp and log-transformed. These ATAC cut-site profiles served as input to k-means clustering (k = 8, range ±800 bp from the dominant TSS).

### H3K27ac ensemble identification

Enhancer ensembles were detected using H3K27ac peaks and mapped reads from the Dome stage (DCC Data IDs: DCD006167DT and DCD008973DT) as input for the ROSE algorithm^58^ with the distance from TSS to exclude, adjusted to 500 bp.

### Genomic coordinate projection

Genomic coordinates of GRB loci were projected between zebrafish and mouse using multiple pairwise sequence alignments between a set of 15 species. The basic concept of our approach is that - under the assumption of conserved synteny - a non-alignable genomic region can be projected from one species to another by interpolating its relative position between two alignable anchor points. The accuracy of such interpolations correlates with the distance to an anchor point. Therefore, projections between species with large evolutionary distances such as zebrafish and mouse tend to be inaccurate due to a low anchor point density. Including so-called bridging species may increase the anchor point density and thus improve projection accuracy. Extended Data Figure 10b illustrates the potential benefit of using a bridging species with a schematic example projection between zebrafish and mouse. The optimal choice of bridging species may vary between different genomic locations and there may be genomic locations for which a combination of bridging species with intermediate projections produces optimal results. Extended Data Figure 10c presents the bridging species optimization problem as the shortest path problem in a graph where every node is a species and the weighted edges between them represent the distance of a genomic location to its anchor point. For that, we established a scoring function that reflects those distances and returns values between 0 and 1, where a score of 1 means that a genomic location x overlaps an anchor point a. The score decreases exponentially as the distance |x − a| increases. For a single species comparison, the function is defined as follows:

$$f\left(x_{i}\right)=\text{exp}\left(-\frac{\text{min}\left(\left|x_{i}-a_{i}^{\left(1\right)}\right|,\left|x_{i}-a_{i}^{\left(2\right)}\right|\right)}{gi\;s}\right),$$

with g denoting the genome size of the respective species and s a scaling factor that can be determined by defining a *distance half-life* d_h_, as the distance |x-a| at which the scoring function is to return a value of 0.5:

$$s=-\frac{d_{h}}{g\;\text{log}\left(0.5\right)}.$$

The length of a path through the graph is then given by subtracting the product of the distance scoring function for every node in the path from 1:

$$l_{p}=1-\prod_{i\in p}f\left(x_{i}\right).$$

The shortest path $\dot{p}$ through the graph is then found by minimizing *l_p_*:

$$\dot{p}={\text{arg}\;\text{min}}_{p\in P}l_{p},$$

with P denoting the set of all paths through the graph. The optimization problem presents a classic shortest path problem and is solved using Dijkstra’s Shortest Path Algorithm^99^.

### Epigenomic Profile Comparison

We compared H3K27me3 ChIP-seq data from phylotypic stages in zebrafish (Prim-5 stage) and mouse (E10.5 stage)^100^, when their transcriptomes are most similar^101^. To match the whole-embryo zebrafish data, we created a virtual embryo dataset for mouse by merging data for 6 different tissues (fore-, mid-, hindbrain, facial prominence, heart, limb). The mouse H3K27me3 profile was projected onto zebrafish genomic coordinates using the multi-species approach by splitting the zebrafish GRB into 1-kb windows, projecting their center coordinates onto mouse, and retrieving the signal from the respective 1-kb bin in mouse. ‘Signal’ stands for H3K27me3 coverage represented as quantiles after quantile normalization of the two distributions in zebrafish and mouse. Signal overlap is represented by the log signal ratio and capped to values in [-1,1]. Signal amplitude represents the maximum signal of zebrafish and mouse to the power of 10 to increase the variance of signal amplitude. For mouse and zebrafish comparison of H3K27ac ensembles previously published data were used^102^.

### Classification of Conservation

Zebrafish ATAC-seq peaks were classified into three conservation classes based on the projection using the multi-species approach. Directly conserved (DC) ATAC-seq peaks overlap a direct alignment between zebrafish and mouse. Indirectly conserved (IC) ATAC-seq peaks do not overlap a direct alignment, but are projected with a score > 0.99, i.e., either overlapping or very close to a multi-species anchor. The remaining peaks are classified as non-conserved (NC). A score of 0.99 means that the sum of the distances from peak to anchor points is < 150 bp considering all intermediate species in the optimal species path.

## Extended Data

## Supplementary Material

Supplementary Information

Supplementary Table 1 - 15

Video 1 - DANIO-CODE DCC Upload Tutorial

Video 2 - DANIO-CODE DCC - Upload data with a .csv file

Video 3 - Add a track from the DANIO-CODE trackhub to the UCSC genome browser

Video 4 - How to use the DANIO-CODE DCC to study stage specific gene expression

Video 5 - How to use the DANIO-CODE DCC to explore regulatory elements and their cell type specifici

## Data Availability

Raw and aligned sequencing data are available at https://danio-code.zfin.org/dataExport/. The raw sequencing data produced for this study are available on the European Bioinformatics Institute (EBI) European Nucleotide Archive (ENA) under study numbers PRJNA824720, PRJNA821001, PRJNA821088, PRJNA821148, and PRJNA821034. Annotation tracks are available at http://genome.ucsc.edu/cgi-bin/hgTracks?db=danRer10&hubUrl=https://danio-code.zfin.org/trackhub/DANIO-CODE.hub.txt (danRer10) and http://genome.ucsc.edu/cgi-bin/hgTracks?db=danRer11&hubUrl=https://danio-code.zfin.org/trackhub/DANIO-CODE.hub.txt (danRer11).

## Code availability

The processing pipelines for the individual assays are available at https://gitlab.com/danio-code. The code used for the analysis is available at https://github.com/DANIO-CODE/DANIO-CODE_Data_analysis (DOI: 10.5281/zenodo.6424702). The script to generate the TrackHub is available at https://gitlab.com/danio-code/TrackHub.
