# Supplementary material for: Multiomic atlas with functional stratification and developmental dynamics of zebrafish cis-regulatory elements
Source: Nat Genet. 2022 Jul 4;54(7):1037–50. doi: 10.1038/s41588-022-01089-w (PMC9279159; doi:10.1038/s41588-022-01089-w)
Supplement: Supplementary file 1 [file 41588_2022_1089_MOESM1_ESM.pdf]

nature portfolio

https://doi.org/10.1038/s41588-022-01089-w

# Supplementary information

# Multiomic atlas with functional stratification and developmental dynamics of zebrafish *cis*-regulatory elements

In the format provided by the authors and unedited

# Supplementary Figures

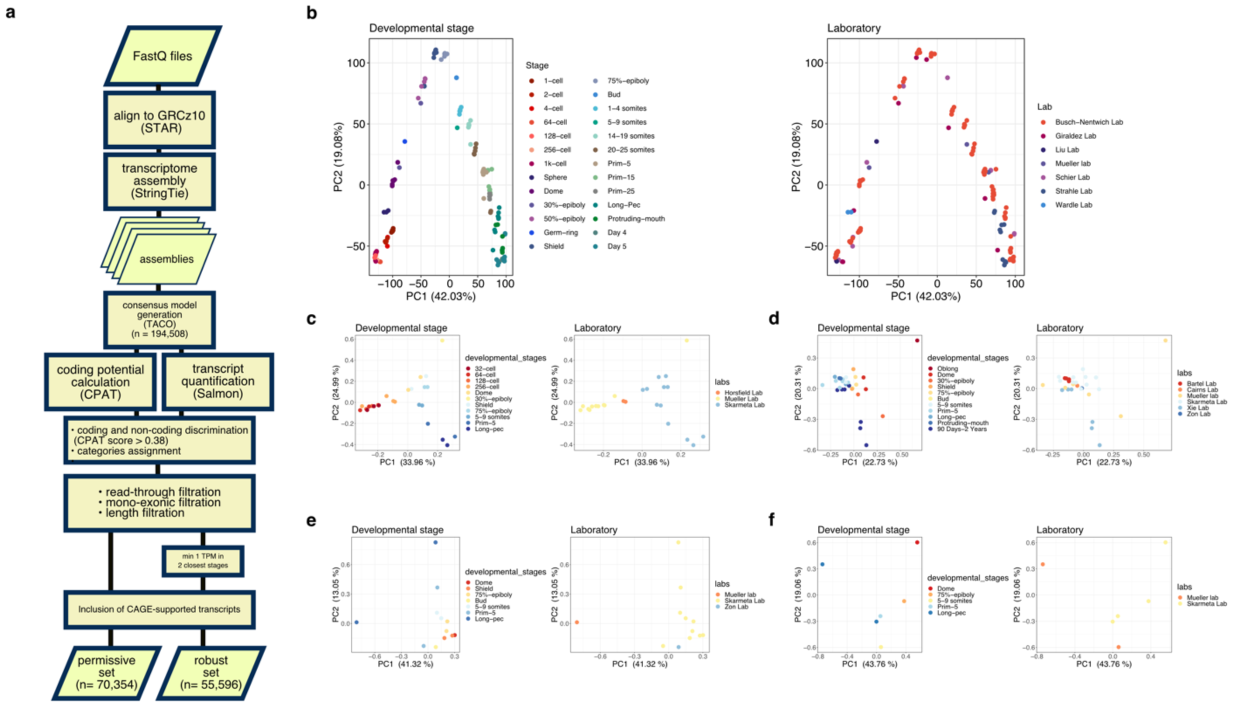


**Supplementary Figure 1. a,** RNA-seq data processing pipeline, **b**, PCA of CAGE-supported RNA seq (n=16,575), labeled by stages (left) and labs (right). PCA of consensus open regions (cPADREs; see **Suppl. Figure 3a**) of **c**, ATAC-seq **d**, H3K4me3 **e**, H3K27ac **f**, and H3K4me1. For H3K4me3 regions were restricted to the promoter-overlapping set.

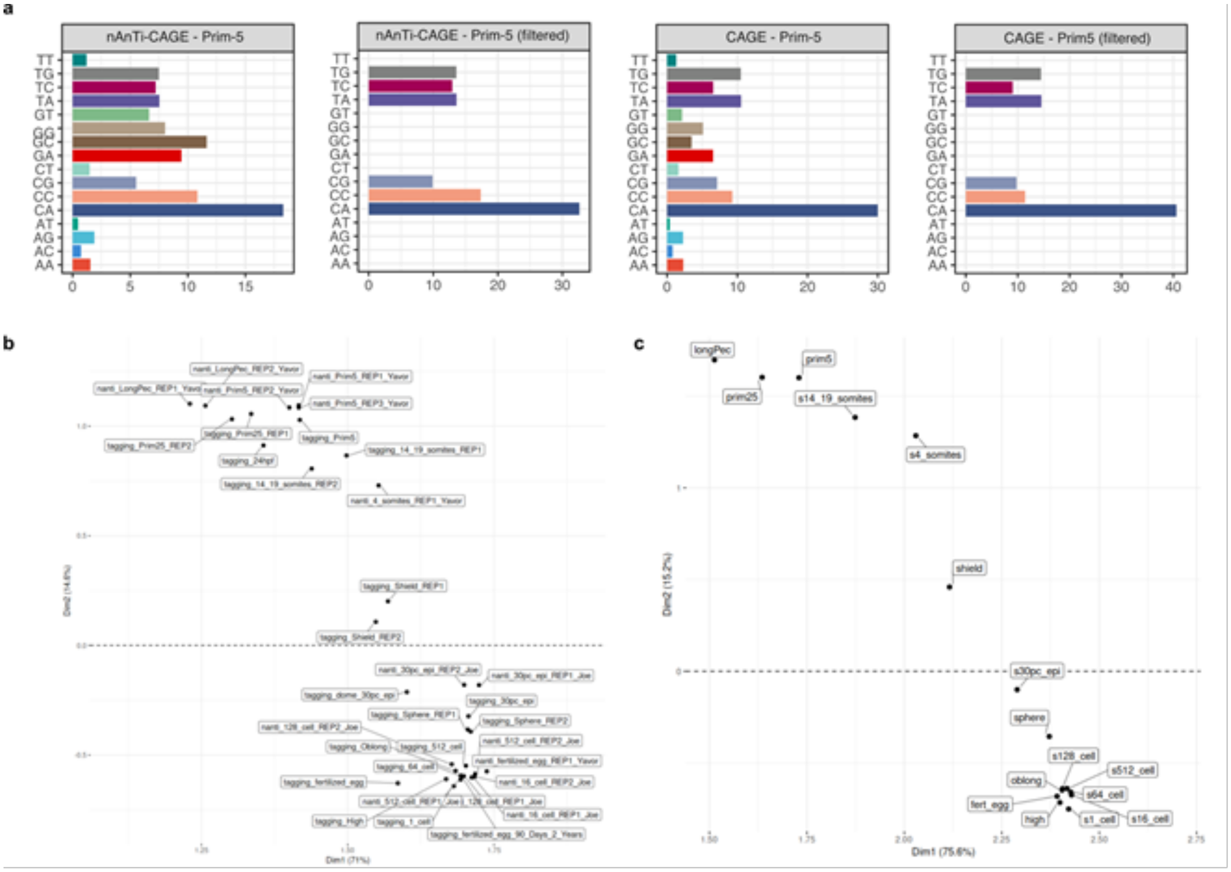


**Supplementary Figure 2. a**, dinucleotide frequency analysis of TSS clusters before and after canonical filtering, **b**, PCA correlation analysis of TSS activity detected by two CAGE protocols, **c**, PCA of 27,781 consensus promoters.

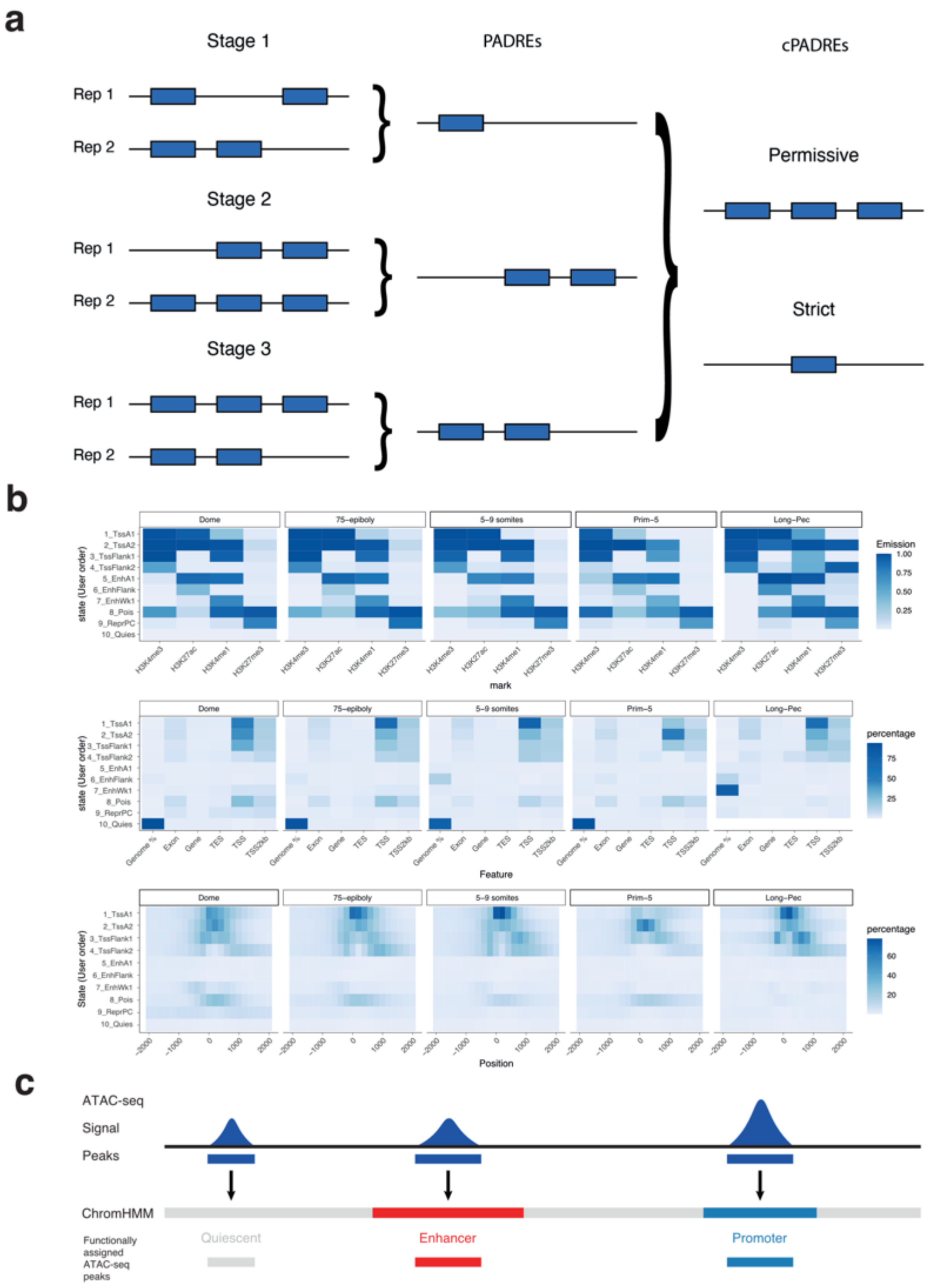


**Supplementary Figure 3. a,** Schematic representation of PADREs and consensus PADREs (cPADREs) definition. PADREs are defined as ATAC-seq peaks reproducible between replicates. We defined cPADREs as a union of PADREs (permissive) and further filtered them with a requirement that a region needs to be open in at least 2 neighbouring developmental stages. The later set was termed a strict cPADRE set and used for the analysis in this paper. **b,** Top: Occurrence probabilities of chromatin marks in each obtained ChromHMM state for five developmental stages. Middle: Enrichment of genomic features in each state for five developmental stages. Bottom: Occurrence of each state +/- 2kb around RefSeq TSS for five developmental stages. The states were manually assigned. 1_TssA1, 2_TssA2 = Active TSS, 3_TssFlank1, 4_TssFlank2 = TSS Flanking region, 5_EnhA1 = Active enhancer, 6_EnhFlank = Weak enhancer, 7_EnhWk1 =

Primed enhancer, 8_Pois = Poised elements, 9_PcRep = Polycomb repressed regions, 10_Quies = Quiescent state. **c,** Functional assignment of PADREs using ChromHMM genome segmentation

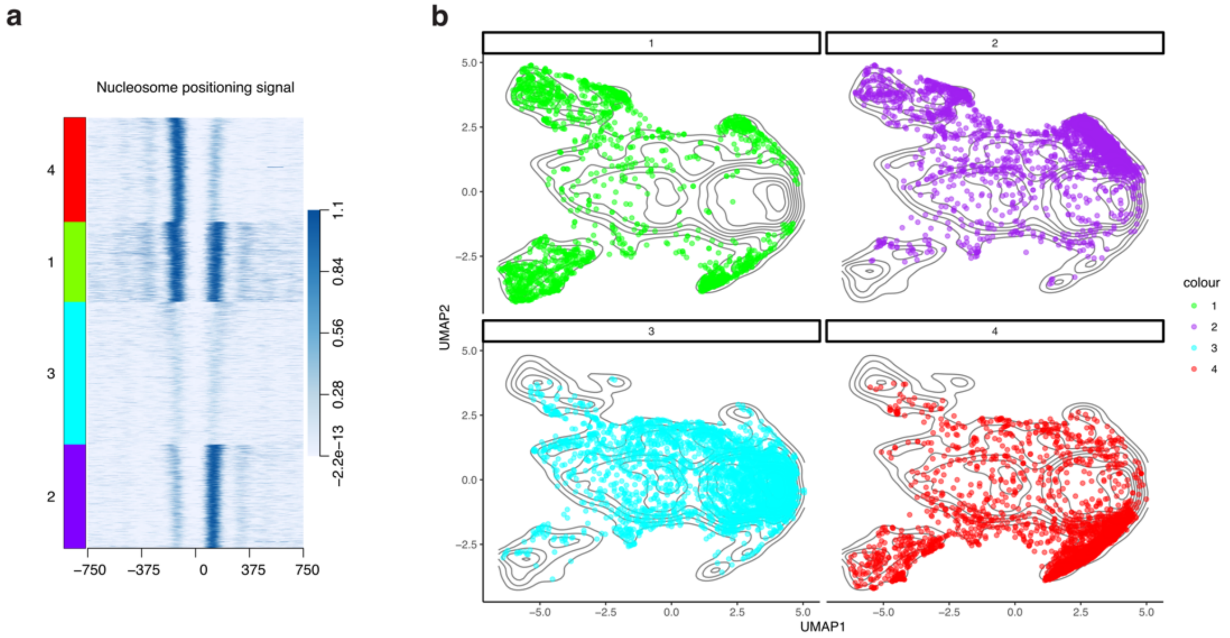


**Supplementary Figure 4.** Nucleosome position around PADREs containing the CTCF motif. **a,** Heatmap with nucleosome position signal PADREs containing the CTCF motif, divided into four groups using the k-means algorithm. **b,** UMAP position of PADREs belonging to groups from **a**.

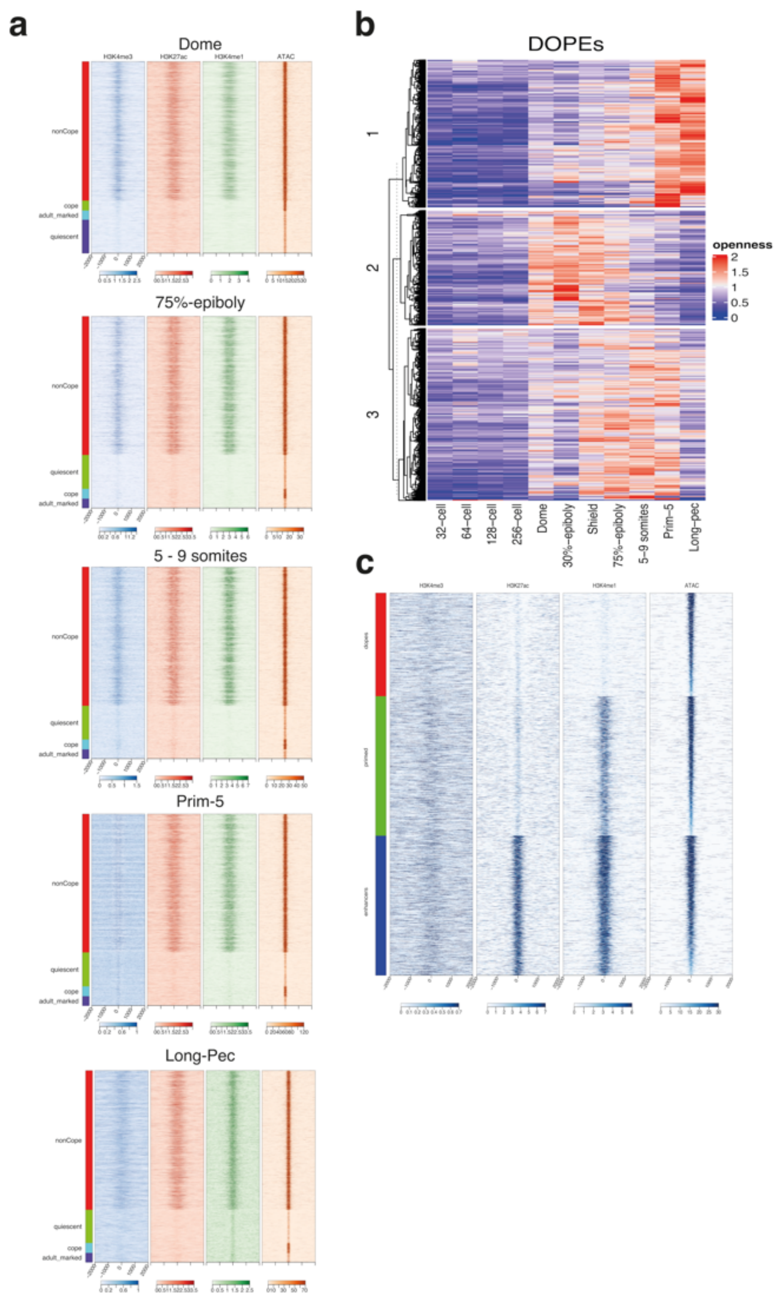


**Supplementary Figure 5. a,** Signal heatmaps of H3K4me3, H3K27ac, H3K4me1, and ATAC for COPEs, DOPEs, DOPEs active in adult tissues, and other constitutive elements throughout development aligned to the centre of open chromatin. **b,** DOPEs and their chromatin openness throughout development. DOPEs were clustered into three groups based on the time of chromatin opening using *k*-means clustering. Signal heatmaps of H3K4me3, H3K27ac, H3K4me1, and ATAC for COPEs, DOPEs, DOPEs active in adult tissues, and other constitutive elements throughout development aligned to the centre of open chromatin. **c,** Signal of active mark around DOPEs with active mark in adult tissue, primed enhancers, and active enhancers with the same range of openness. The lack of active marks in DOPEs with comparable ATAC-seq signal with other two classes shows that this phenomenon is unluckily due to the regulatory elements being active only in rare cell types but reflects the existence of a novel class of regulatory elements that

exist in a state without most common marks associated with active enhancers. This might indicate the existence of a yet unrevealed mechanism of enhancer activation.

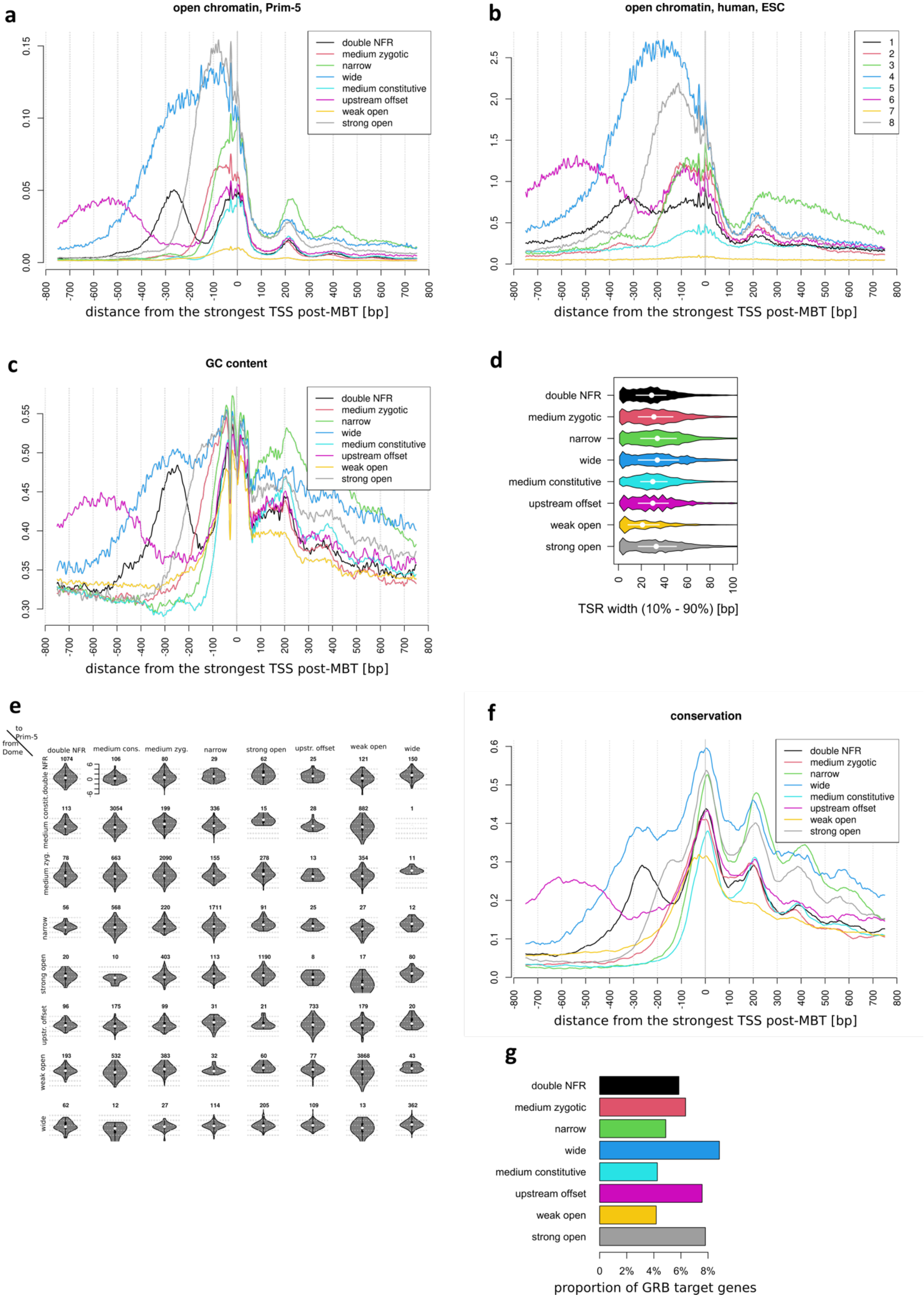
a
open chromatin, Prim-5
double NFR
medium zygotic
narrow
wide
medium constitutive
upstream offset
weak open
strong open
distance from the strongest TSS post-MBT [bp]
b
open chromatin, human, ESC
1
2
3
4
5
6
7
8
distance from the strongest TSS post-MBT [bp]
c
GC content
double NFR
medium zygotic
narrow
wide
medium constitutive
upstream offset
weak open
strong open
distance from the strongest TSS post-MBT [bp]
d
double NFR
medium zygotic
narrow
wide
medium constitutive
upstream offset
weak open
strong open
TSR width (10% - 90%) [bp]
e
to Prim-5
from Dome
double NFR
medium cons.
medium zyg.
narrow
strong open
upstr. offset
weak open
wide
f
conservation
double NFR
medium zygotic
narrow
wide
medium constitutive
upstream offset
weak open
strong open
distance from the strongest TSS post-MBT [bp]
g
double NFR
medium zygotic
narrow
wide
medium constitutive
upstream offset
weak open
strong open
0
2%
4%
6%
8%
proportion of GRB target genes

**Supplementary Figure 6. a**, Aggregated (mean) chromatin accessibility profiles plotted against the dominant transcription start site. **b,** Aggregated (mean) chromatin accessibility profiles in human embryonic stem cells. k-means clustering reveals very similar clusters as in zebrafish (a). **c**, Promoters of each class are enriched in GC content. GC content stays high until +50bp when it abruptly switches into the WW oscillating region (nucleosome positioning signal). The fine structure of nucleosome positioning signal around +50bp to +200bp is similar in each class. The nucleosome positioning oscillation is diminished around the nucleosome dyad (+120bp), and then it reappears in the second half of the nucleosome-wrapped fragment. **d**, Transcription start region (TSR) width in base pairs measured by CAGE signal distribution, incorporating the central 80% of CAGE expression. **e**, Expression dynamics of promoters transitioning between classes at the Dome and Prim-5 stages. Violin plots show expression log-fold changes. **f,** Aggregated teleost sequence conservation (phastCons). Sequence conservation always peaks at the TSS. There are additional conservation peaks visible downstream between nucleosome linker regions. In three cases where there are open regions upstream, conservation follows openness upstream. Generally, conservation follows openness, suggesting purifying selection acting on regulatory sites. **g,** Bar chart of proportion of promoter classes containing target genes of Gene Regulatory Block (GRBs, see text associated with Figure 5).

# Supplementary Methods

## *Data Processing*

Sequencing files from the DANIO-CODE DCC were processed with standardised pipelines, see https://gitlab.com/danio-code for details. The resulting files were uploaded to the DCC annotated with the version of the used pipeline and linked to their source files. RNA-seq, methylation data, HiC, 4C-seq data were processed using custom processing pipelines. ChIP-seq and ATAC-seq data were processed using adapted ENCODE pipelines[1] and CAGE-seq and 3P-seq data were processed using adapted FANTOM pipeline[2].

## *WashU Epigenome Browser tracks*

In order to visualize the developmental timeline, bigwig files of the different assays were combined to one matplotlib track and each track was shifted by adding a fixed amount to their score column. To improve readability, the empty regions were set to a score of 0 before the shift. Furthermore, each assay was scaled to reduce noise. The code and the session file are available on GitHub https://github.com/DANIO-CODE/DANIO-CODE_Data_analysis/tree/figure_1/Figures/Figure1.

Zebrafish developmental stage drawings were adapted from Kimmel et al., 1995[3].

The drawing of the adult stage in **Figure 1** was created with BioRender.com.

## *Track hubs*

Processed data track can be uploaded to UCSC Genome browser as a track hub[4]. The DCC track hub was generated using custom scripts and it is available in the following link: https://danio-code.zfin.org/trackhub/DANIO-CODE.hub.txt, but also registered at UCSC as a public track hub. Annotation data are also available in the track hub available in the following link: http://trackhub.genereg.net/DANIO-CODE_Functional_annotation_tracks/hub_test/hub1.txt

## *Box plot definition*

Box plots throughout the manuscript were defined in the standard way with the center depicting the median, the hinges of the box depicting lower and upper quartiles, and the whiskers extending

1.5 * inter-quartile width from the lower and upper box hinges. The outliers were depicted as points.

## *Nanti-CAGE-seq on zebrafish developmental stages*

Total RNA was extracted from multiple stages of zebrafish development (Fertilized egg, 16-cell, 128-cell, 512-cell, 30%-epiboly, 4 somite, Prim-5, and High-pec) using the miRNeasy kit (Qiagen), according to the manufacturer's instructions. nAnT-iCAGE libraries were prepared as described previously[5] using the CAGE™ Preparation Kit (DNAFORM). All libraries were sequenced on Illumina HiSeq2500 except the high pec library which has been sequenced on NextSeq500.

## *Total and Nuclear Tagging CAGE-seq*

Total RNA was collected from multiple stages of zebrafish development (30%-epiboly, 9 somite, Prim-5, High-pec and Larvae) using the miRNeasy kit (Qiagen), according to the manufacturer's instructions. Nuclear RNA was also collected, through a nuclear isolation protocol based on the Nuclei EZ isolation kit (Sigma) but optimized for zebrafish tissue. In summary, embryos were de-chorionated and then dissociated and de-yolked in a cell swelling buffer (250mM Sucrose, 10mM Tris-HCL (pH 7.9), 10mM $MgCl_2$, 1mM EGTA), through vigorous pipetting. Embryo slurry was left to stand for 5 minutes and then filtered using a 50uM tube top filter. It was then centrifuged at 500 g, 5 mins, 4°C and the pellet resuspended in Freezing buffer (Sigma), after which they were stored at -80°C. Upon defrosting Nuclei EZ Lysis Buffer was added to the cells, vortexed and left to stand for 5 mins. Nuclei were then centrifuged at 500 g, 5 mins, 4°C and the pellet washed once in Nuclei EZ Lysis Buffer. Upon a final centrifugation of the nuclei, the pellet was resuspended in Qiazol Lysis Reagent (Qiagen) and RNA extracted using the miRNeasy kit (Qiagen), according to the manufacturer's instructions. Tagging-CAGE libraries were prepared from both the total and nuclear fractions following the protocol described previously[6]. All libraries were sequenced on Illumina HiSeq2500 by the SNP&SEQ Technology Platform in Uppsala, Sweden.

*Embryo preparation and ChIP experiments*

Approximately 1500 Dome/30%-epiboly, 25 5-9 somites and 200 Prim-5/Long-pec embryos from natural crossing of wild type AB strain adults were collected. The embryos were enzymatically dechorionated using pronase and fixed in 1.85% formaldehyde in Hanks media for 20 minutes at room temperature. After crosslinking, samples were washed once with PBS and the fixation was stopped by incubating in 1x glycine for 10 minutes at room temperature followed by three washes with ice-cold PBS.

The embryos at somite stages were fixed in 1% formaldehyde for 10 minutes at room temperature upon dissociation in 1 ml of PBS and 1x protease inhibitor cocktail. Fixation was stopped with 115 µl of glycine for 5 minutes at room temperature. The cell suspension was centrifuged at 300 g for 10 minutes at 4°C and pellets were washed with Hank's balanced salt solution (HBSS) supplemented with 1x protease inhibitor cocktail (PIC) and centrifuged at 300 g for 10 minutes. Supernatants were removed and pellets were kept on ice.

ChIP experiments were carried out using the ChIP-IT Express Enzymatic kit (Cat. No. 53009, Active Motif) or the True MicroChIP Kit (Cat. No. C01010130, Diagenode) for the 5-9 somite stage embryos. Both protocols were applied in line with manufacturer's instructions.

Briefly, for the ChIP-IT Express Enzymatic Kit, crosslinked embryos were resuspended in 1 ml ice-cold lysis buffer, incubated on ice for 20 minutes, transferred to a pre-cooled dounce homogenizer and dounced by 10 strokes. Nuclei were collected by centrifugation, resuspended in 200 µl digestion buffer and incubated at 37°C for 5 minutes. Chromatin was sheared by adding 10 µl of enzymatic shearing cocktail working stock (200 U ml−1) and incubating for 10 minutes at 37°C. Shearing efficiency was checked by gel electrophoresis according to manufacturer's instructions. The reaction was stopped by adding 5 µl ice-cold 0.5 M EDTA and incubating on ice for 10 min and sheared chromatin was cleared by centrifugation. For ChIP reactions 70 µl of sheared chromatin were mixed with 25 µl Protein G magnetic beads, 20 µl ChIP buffer 1, 1 µl protein inhibitor cocktail and 4 µg of antibody or an equivalent volume of water (no antibody control), respectively, and water to a final volume of 200 µl. ChIP was performed in duplicates for each stage. ChIP reactions were then incubated in rotation overnight at 4°C. Magnetic beads were washed and incubated in elution buffer. After addition of reverse crosslinking buffer samples were

de-crosslinked for 4 hours at 65°C. Samples were treated with Proteinase K and RNase A and purified using phenol chloroform extraction.

ChIPs for 5-9 somite stage embryos were performed with the True MicroChip in crosslinked and dissociated embryos as described above. Cell pellets were incubated with 25 µl lysis buffer and 1x PIC for 10 minutes on ice. Upon addition of 75 µl of HBSS the cell suspension was sonicated with 12 cycles (30 seconds ON/ 30 seconds OFF) using a Bioruptor Pico sonicator. Sonicated samples were centrifuged at 14000 g for 10 minutes and the supernatant was mixed with 100 µl of tC1 buffer and 1x PIC. Chromatin was incubated with the antibody in rotation overnight at 4°C together with 11 µl of pre-washed. Chromatin-bound beads were captured using a magnetic rack and washed with 100 µl tW1, tW2, tW3 and tW4 buffer respectively, for 4 minutes at 4C each time. Beads were resuspended in elution buffer tE1 and incubated for 30 minutes at room temperature. Finally, supernatants were transferred to clean tubes and de-crosslinked with 8 µl of Elution buffer tE2 for 4 hours at 65C. DNA was purified using Micro ChIP spin columns.

Antibodies used for ChIP-seq with dilutions and reference are as following: H3K4me3 (1:40, C15410003 (Diagenode)), H3K27ac (1:30, ab4729 (abcam)), H3K4me1 (1:30, ab8895 (abcam)), H3K27me3 (1:50, C15410069 (Diagenode)), H3K36me3 (1:30, ab9050 (abcam)), Pol II (1:30, ab5131 (abcam)), and H2A.Z (1:30, ab4174 (abcam)).

### *Library preparation for ChIP-seq experiments*

The DNA libraries were prepared using the Microplex library preparation Kit V2 (Diagenode, Cat. No. C05010013) following the manufacturer's instructions. Briefly, templates were prepared with 2 µl of template preparation buffer and 1 µl of preparation enzyme and the reaction was carried in a thermocycler as follows: 25 minutes at 22ºC, 20 minutes at 55ºC and hold at 4ºC. Upon template preparation libraries were synthesized with 1 µl of library synthesis buffer and 1µl of library synthesis enzyme incubate the samples at 22ºC for 40 minutes before proceeding to library amplification. Finally, libraries were amplified adjusting the number of cycles to the amount of DNA used for the preparation, according to the instructions. All libraries were purified and size selected with AMPure XP beads (Beckman Coulter) using the ratios recommended by the microplex protocol. The quality and concentration of the samples was verified by High Sensitivity D5000 ScreenTape (Agilent) and Qubit dsDNA High sensitivity assays (Thermo fisher scientific).

### *ATAC-seq experiments*

Chromatin was prepared using 200 embryos, 100 embryos and 50 embryos in the 32-cell, 128-cell and 256-cell stages, respectively. Embryos were de-chorionated enzymatically using pronase and collected in 1.5ml Eppendorf tubes. 1ml Cell Dissociation Buffer (13151014, Gibco) was added into the tubes and cells were dissociated by pipetting up and down several times. Dissociated cells were then collected by spinning at 500 g for 10 min at 4°C. The following steps followed the ATAC-seq protocol as previously published[7].

### *Cell preparation for Hi-C*

Samples were prepared as previously described[8,9]. Briefly, Tg(Buc-GFP) heterozygous embryos were dissociated in 500 ml of HBSS supplemented with 0.25% BSA and 10 mM Hepes by pipetting for 2 minutes with a glass pipette. Excess of yolk was removed by two rounds of 3 minutes centrifugation at 350 x g at 4ºC, while pelleted cells were resuspended in 1 ml HBSS supplemented with 0.25% BSA and 10mM Hepes prior to filtering. The cell suspension was kept on ice during sorting in a FACS Aria Fusion sorter.

### *Low input somatic cells in situ Hi-C library generation*

Briefly, we performed low input in situ Hi-C[9] with small modifications on sorted somatic cells in biological duplicates. 6,000 FACS-sorted somatic cells were crosslinked in 1% formaldehyde, incubated for 10 min at room temperature with rotation (20 rpm) and quenched by adding 0.2M glycine for 5 min at room temperature with gentle rotation (20 rpm). Cells were then washed three times with 1 mL cold PBS (centrifuged at 300 g for 5 min at 4C) and lysed using ice-cold in situ Hi-C buffer (10 mM Tris-Cl pH 8.0, 10 mM NaCl, 0.2% IGEPAL CA-630, cOmplete Ultra protease inhibitors) by incubating them on ice for 15 min. Centrifuged nuclei were then resuspended in 125 mL ice-cold NEB2 buffer, pelleted (13,000 g for 5 min at 4°C) and permeabilized in 12.5 mL of 0.4% SDS at 65C for 10 min. SDS was quenched by adding 12.5 mL of 10% Triton X-100 and 85 mL of nuclease-free water and incubating at 37°C for 45 min with shaking. Chromatin was digested with 100 U of MboI restriction enzyme (90 min at 37°C with rotation; New England Biolabs) and the enzyme was heat-inactivated at 62°C for 20 min. Digestion generated overhangs were filled in with a mixture of 0.4 mM biotin-14-dCTP (10 mL; Thermo

Fisher Scientific) and 10 mM dATP/dGTP/DTTP (0.5 mL each; Thermo Fisher Scientific) by incubating with DNA polymerase I Klenow (4 mL; New England Biolabs) for 90 min at 37°C with rotation. DNA fragments were ligated using T4 DNA ligase (Thermo Fisher Scientific) and a mix (60 mL of T4 DNA ligase buffer, 50 mL of Triton X-100, 6 mL of BSA 20mg/ml, 3.5 mL of T4 DNA ligase and 328.5 mL of nuclease-free water) during 4 h at 20°C with gentle rotation. Nuclei were gently pelleted (2,500 g for 5 min at room temperature) and resuspended in 250 mL extraction buffer. Protein was digested with 10 mL Proteinase K (20mg/ml; Applichem) for 30 min at 55°C with shaking (1,000 rpm) followed by adding 65 mL of 5M NaCl and an overnight incubation at 65°C with shaking (1,000 rpm). Phenol-Chloroform-Isoamyl alcohol (25:24:1; Sigma-Aldrich) DNA extraction was performed on the nuclei and final DNA was resuspended in 25 mL of 10 mM Tris pH 8.0 (Applichem) and incubated with RNase A (1 mL of a 10 mg/mL stock; Applichem) for 15 min at 37°C. Biotin removal from unligated fragments was performed by incubating DNA samples for 4h at 20°C in a mix of 5 mL of 10X NEB2 buffer (New England Biolabs), 5 mL of a 1mM dNTPs mix, 0.25 mL of 20 mg/ml BSA, 3.5 mL of T4 DNA polymerase (New England Biolabs) and up to 50 mL of nuclease-free water. Samples were then sheared using a Covaris S220 instrument (2 cycles, each 50s, 10% duty, 4 intensity, 200 cycles/burst). Dynabeads MyOne Streptavidin C1 beads were used to pull down biotinylated fragments. NEBNext Ultra End Repair and the NEBNExt Ultra dA-Tailing modules were used for end repair and adding Illumina sequencing adaptors. Final PCR amplification was done in 4 parallel reactions per sample (10 mL of the bead-bound libraries, 25 mL of 2x NEBNext Ultra II Q5 Master Mix, 3 mL of 10 mM Universal PCR primer from Illumina, 3 mL of 10mM Index PCR primer and 9 mL of nuclease-free water and amplified for 10 cycles (98C for 10 s, 65°C for 75 s, ramping 1.5°C/s for 10 cycles and a final step at 65°C for 5 min). The four reactions were combined into one tube and size selected using Ampure XP beads (Beckman Coulter). Final Hi-C libraries were quantified using Qubit dsDNA HS assay kit and a DNA HS kit on a 2100 Bioanalyzer (Agilent). Libraries were first shallow sequenced on an Illumina MiSeq (2x84bp paired-end; MiSeq reagent kit v3-150 cycles) to assess library quality. Finally, libraries were deeply sequenced on an Illumina NextSeq (2x80 bp paired-end; NextSeq 500/550 High Ouput kit v2-150 cycles).

## 4-C data generation

4C-seq experiments were performed and analysed as described earlier[10]. For zebrafish, 500 embryos at the 24hpf stage were dechorionated with pronase and deyolked using the Ginzburg Fish Ringer buffer (55 mM NaCl, 1.8 mM KCl and 1.25 mM NaHCO3). Then they were fixed using 2% PFA for 15 minutes at room temperature. Fixations were stopped by adding glycine and washing several times with PBS. Then, the fixed samples were lysed (lysis buffer: 10 mM Tris-HCl pH 8, 10 mM NaCl, 0.3% Igepal CA-630 (Sigma-Aldrich, I8896) and 1x protease inhibitor cocktail (Complete, Roche, 11697498001)), and the DNA was digested with DpnII (New England BioLabs, R0543M) and Csp6I (Fermentas, Thermo Scientific, FD0214) as primary and secondary enzymes, respectively. T4 DNA ligase (Thermo scientific, EL0014) was used for both ligation steps. Specific 4C-seq primers containing single-end Illumina adaptors were designed using as bait the different promoters of interest. For each library, eight independent PCRs were performed using the Expand Long Template PCR System (Roche, 11759060001) that were subsequently pooled and purified using AMPure XP beads (Beckman Coulter, A63883). Then libraries were sent for single-end sequencing. 4C-seq sequencing reads were aligned to the zebrafish reference genome using bowtie. Reads located in fragments flanked by two restriction sites of the same enzyme, in fragments smaller than 40 bp or within a window of 10 kb around the viewpoint were filtered out. Mapped reads were then converted to reads per first enzyme fragment ends and smoothened using a 30-fragment mean running window algorithm. This signal is ready to be visualized. More details and supporting code are available in the GitLab repository (https://gitlab.com/danio-code/danio-code_4c-seq).

## Guide RNA preparation and microinjection

Single guide RNAs (dried pellet, Agilent) were resuspended with $H_2O$ into 100 mM stock concentration. Zebrafish codon optimized dCas9-Neon was cloned into pCS2+ vector, then the plasmid was linearized with NotI and dCas9-Neon mRNA was synthesized in vitro with mMESSAGE mMACHINE™ SP6 Transcription Kit (Ambion). Single guide RNA (80 nM/ul) and dCas9neon mRNA (500 ng/ul) were injected into newly fertilized one cell stage zebrafish embryos at 2 nl volume.

### *KEGG pathway enrichment analysis*

KEGG Pathways enrichment analysis of alternative promoter usage was performed using the Enrichr and FishEnrichr tools[11]

## *Genome browser visualisation of chromatin marks and annotated regions*

Chromatin marks and gene tracks (**Figure 3A**, **Figure 5C)**, as well as the corresponding annotation tracks (ChromHMM, PADREs, and ensembles) were visualized with the Gviz package[12].

## *Validation of PADREs by intersection with enhancers studied by transgenesis.*

A publication search was conducted in PUBMED (https://pubmed.ncbi.nlm.nih.gov) by Boolean term combinations including variations of the search terms "zebrafish, transgenic, enhancer, conserved regulatory element, NOT ATAC, NOT ChIP" on 30/06/19. Publications were considered for further analysis if the initial transgenesis was based on prediction by sequence similarity, transcription factor binding site, TSS prediction and deletion assays where fragments were tested blindly and reduced to the functional unit after deletions. Publications, in which the enhancers presented were based on chromatin feature predictions and no transgenesis was performed and where the direction of the study was from genomics data toward locus determination then followed by transgenesis were disregarded. In addition, a small number of manual collection of enhancers of *ngn1*, *id1* and *sox9* were added from co-authors' publications to the list. Anatomical terms associated with transgene expression together with chromosomal locations of individual enhancers were extracted from the publications and included in a custom UCSC Genome Browser track created for the GRCz10/danRer10 and GRCz11/danRer11 assemblies in extended BED format. Transgenically identified enhancers were used for validation of PADREs by intersection with them.

## *eRNA calling*

We identified eRNA using CageFightR v1.8.0[13] on the 24 total and nuclear Tagging CAGE-seq samples with the standard parameters. We applied an expression filter, keeping only clusters with an expression greater than zero (unexpressed=0) in more than two samples (minSamples=2).

Furthermore, only intergenic and intronic clusters were kept. The code is available in the GitHub repository.

### *Identification of CTCF binding sites*

The CTCF PWM matrix was taken from the JASPAR database[14] under the ID MA0139.1. PADREs were scanned for CTCF motif using TFBSTools[15], using a threshold of 90% identity.

### *Conserved Non-Coding Elements (CNEs) and phastCons scores*

CNEs used were downloaded from Ancora (http://ancora.genereg.net)[16]. Human CNEs used were 70% matched over 50 columns, and Mexican cavefish CNEs used were 90% matched over 50 columns.

For the conservation analyses, the cyprinid (grass carp, common carp, goldfish, and zebrafish) phastCons[17] score from Chen *et al.*[18] were used. The data was downloaded from the NHGRI ZebrafishGenomics track hub (link to the track: https://research.nhgri.nih.gov/manuscripts/Burgess/zebrafish/downloads/NHGRI-1/danRer10/ZV10tracks/ZF_GC_CC_GF.bw), listed in the UCSC Public Hubs list.

The conservation tracks for the genome versions danRer10/GRCz10 and danRer11/GRCz11 were mirrored in the public DANIO-CODE track hub with the authors' permission.

### *Classifications of distal elements by chromatin opening dynamics throughout development*

Distal cPADREs were filtered by excluding cPADREs +/- 500bp around CAGE-defined promoters. For each distal cPADRE the fold change of the ATAC-seq signal over background was calculated at every developmental stage. Each distal cPADRE were normalized stage-wise by dividing it's value by root-mean-square and those values were used as input for the self-organizing maps (SOMs)[19]. SOMs were performed using the kohonen package[20]. The clustering was performed using a 4x4 hexagonal grid, which resulted in 16 classes ordered in a 4x4 grid. The ATAC-seq, H3K27ac ChIP-seq, and H3K4me1 ChIP-seq signals of elements assigned to classes

4, 6, and 14 were visualized with the genomation package[21] with a window of +/-2000bp around the peak summit. Peak summits for each stage were defined with the refinepeak command from the MACS2 program package[22] using the Dome and Prim-5 stage-specific mapped reads.

### *DNA Methylation analysis*

Bisulfite-converted (WGBS) sequence reads from developing zebrafish embryos[23-25] were trimmed using Trimmomatic with the following settings: ILLUMINACLIP:TruSeq3-SE.fa:2:30:10 SLIDINGWINDOW:5:20 LEADING:3 TRAILING:3 MINLEN:20[26]. Using Walt (-m 5 -t 20 -N 10000000)[27], trimmed reads were mapped onto bisulfite-converted reference genomes for both GRCz11 and GRCz10 (UCSC) with the λ genome added as a separate chromosome in order to estimate bisulfite conversion efficiencies. The resulting SAM files were converted to BAM format and were deduplicated using sambamba markdup[28]. Due to some samples containing high levels of non-conversion rates (>1%), BAM files were filtered to remove reads containing more than 3 non-converted cytosines outside the CG context using samtools[29], picard (http://broadinstitute.github.io/picard/) and a custom awk script. Methylation levels were called using MethylDackel (extract --mergeContext) (https://github.com/dpryan79/MethylDackel), and Bigwig files were generated from the resulting bedGraph files using UCSC kentUtils command bedGraphToBigwig[30]. Matrices for heatmaps were generated using Bigwig files and deepTools[31] (computeMatrix reference-point -b 2500 -a 2500 -bs 25). NAN values from the resulting Matrix files were replaced with zeroes and heatmaps were plotted using deepTools plotHeatmap. Average methylation levels for chromHMM analyses were calculated using BEDtools[32] (map -o sum) by dividing the sum of reads supporting a methylated CG by the number of reads mapped to that region.

### *Cell-type specificity assignment*

Genome-wide cell-type specificity of the genome version danRer11 was assigned from single-cell ATAC-seq data using the scregseg-pi algorithm described in McGarvey *et al.*[33]. In brief, the scregseg-pi annotations are based on a hidden Markov model (HMM) that takes as input ATAC-seq read counts across cell-type clusters in 500 bp bins across the genome. An HMM was fitted with 30 states that describe the cross-cell type accessibility profile using Dirichlet multinomial

emission probabilities. The HMM was employed to group the genomic regions into states with similar accessibility profiles. Broadly speaking, three types of states were identified: 1) states that reflect accessibility in individual cell types, 2) states that represent accessibility in multiple cell types and 3) background states that reflect genomic background noise. By investigating state summary statistics and comparing state call proximity to cell-type marker genes (e.g., marker genes derived from Zfin and corresponding scRNA-seq[34]), 17 foreground states were identified. The remaining 13 states represented genomic background. For each genomic 500 bp the state with maximal posterior probability was assigned. Prim-5 PADREs were converted from the danRer10 version to danRer11 using the liftOver function from the rtracklayer package[35]. Cell-type of PADREs was determined by overlapping with scregseg-pi segments which state was defined by maximal posterior probability.

The anatomical terms of transgenically validated enhancers were compared to the cell-type specificity annotations of PADREs active at the Prim-5 stage published in McGarvey *et al.*, 2022 for assessment of overlap. If an anatomical term or tissue registered in the publications as activity domain for the transgenes was identical or was part of a cell-type or tissue assigned by McGarvey *et al.* an overlap was registered.

While the PADRE assignment of all 17 foreground states is present in the DANIO-CODE track hub, we show the UMAP profiles and signal profiles of three states (5, 10, and 29). We termed state 5 as neural precursors, because of the cell-types they are active in and marker genes they express as shown in McGArvey *et al.* Similarly, we termed state 10 as muscle. State 29 was specific for only one cell-type annotated as differentiating neurons in McGarvey *et al*. To account for the cell number variation in the whole embryo data, we compared the intensities of signals between cell-types only when signals from independent assays could be used as reference (e.g., neural progenitors and muscle have different openness profiles, but the same level of H3K27ac and H3K4me1) or when the ratio between signals of different assays within one cell-type is different from those in other cell-types (e.g. H3K27ac level in differentiating neurons compared to other marks is different of those in nerual progenitors). The chain file for lift-over was downloaded from the UCSC genome browser[36]. The ATAC-seq, H3K27ac ChIP-seq, and H3K4me1 ChIP-seq signals of neural precursors, muscle, and differentiating neuron specific

elements were visualized with the genomation package[21] with a window of +/-750bp around the peak summit.

### *Expression clustering*

We grouped 27,781 consensus clusters based on their expression log fold changes using self-organising maps choosing a 5 x 5 grid as in previous work[37]. These analyses separated consensus clusters mostly by maternal and zygotic expression. Afterwards we manually grouped the resulting 25 cells into maternal (blue), zygotic (red), ZGA-peaking (brown) and constitutively expressed at distinct levels (gray).

### *Identification of topologically associated domains (TADs)*

Long-pec (48 hpf) Hi-C matrices (Hernandez-Rodriguez, Díaz, et al., Locus-specific functional conservation of chromatin structure across vertebrate evolution. In revision) were mapped to the GRCz10 genome and processed using FAN-C v0.9.0 with default parameters[38]. Merged matrices were binned at 10kb resolution. TADs were interactively called using TADtool[39] with a window size of 110 kb and a cut-off of 0.004148 over the insulation index signal.

### *Identification of Genomic Regulatory Blocks (GRBs)*

The GRBs used were called from whole genome alignment between zebrafish and mexican cavefish as described previously[40], with the following parameters: 70% match and 18kb window. Extreme conservation is a (fat) tail of a continuum, so some "non-GRB TADs" might be TADs with higher turnover of noncoding conservation. Our comparison looked for enrichments, not sharp differences.

### *Hi-C data processing and normalisation.*

Raw sequencing reads were processed using HiCUP v0.6.0[41]. Sequencing reads were mapped against the danRer10 genome, with Bowtie2 v2.3.4.1[42] as the aligner. Experimental artifacts, such as circularised fragments or re-ligations, were filtered out, and duplicate reads were removed. The aligned Hi-C data were normalised (coverage-corrected) using a matrix balancing algorithm by HOMER v4.11[43].

## *Aggregate contact map analysis around H3K27ac ensembles*

Ensembles were categorised as GRB or non-GRB ensembles, and 50-150 kb long ensembles were selected for this analysis. Control sets of ensembles were generated by shifting the ensemble coordinates by 10 mb, or randomly uniformly sampling GRB/non-GRB TADs with/without ensembles. All sets were downsampled to the smallest set. The aggregate coverage- and distance-corrected Hi-C maps around these ensemble sets were computed at 10 kb resolution in a +/-500 kb or +/-150 kb window around the centre of ensembles, using HOMER's analyzeHiC function with the options -hist 10000 -superRes 10000 -norm -normTotal given -normArea given.

## *Compartment analysis.*

The compartment signal was computed as the first principal component of the normalised (coverage- and distance-corrected) interaction profile correlation matrix at 50 kb bin resolution using the runHiCpca.pl function of HOMER. Chromosomes where the contact enrichment within chromosome arms was stronger than the compartment signal, we used the second principal component. Chromosomes 4 and 7 were excluded from the analysis as the compartment calls were poor quality due to assembly issues. TADs were categorised into 4 categories, based on whether they were GRB or non-GRB TADS and whether they did or did not contain ensembles. For all TADs, a compartment score was defined as the compartment signal of the central 50 kb bin of the TAD. The distributions of compartment scores for the GRB/non-GRB TADs with/without ensembles were compared using two-sided two-sample unpaired Wilcoxon test.

## *Expression profiles of genes in TADs with H3K27ac ensembles*

CAGE promoters located in TADS with H3K27ac ensembles are classified as H3K27ac associates if their promoter is located within the ensemble or in the 12.5kb flanking region of the ensemble. The expression of each promoter in H3K27ac TADs throughout development was centered and scaled, and the promoters were clustered using the kohonen package[20]. The clustering was performed using a 3x3 hexagonal grid, which resulted in 9 classes ordered in a 3x3 grid. The obtained classes were visualized using the ComplexHeatmap package[44].

## *Expression of target and bystander genes*

The list with prediction of human target and bystander genes was used from Tan, 2017. Zebrafish orthologs were identified using a homology table from biomaRt[4,45]. To account for ohnologs, only

predicted target genes with a homolog in a GRB TAD were considered in the analysis. The expression of each promoter in GRB TADs throughout development was centered and scaled, and the expression was visualized using the ComplexHeatmap package.

### *H3K27ac signal across TADs*

H3K27ac signal across TADs was calculated using the ScoreMatrixBin function from the genomation package with 400 bins. Signals of H3K27ac for each stage were used as targets. For windows, each TADs were ordered by length and each TAD was extended to the size of the largest TAD. The calculated matrices were smoothed and visualized using the heatmaps package (Perry, 2021, doi: 10.18129/B9.bioc.heatmaps).

### *Motif search for regulatory element projection*

Motif for cross-species comparison hits were computed using seqPattern on a set of position weight matrices for 120 transcription factor families. Regions with scores of >85 were considered as motif hits.

## Supplementary References


1. Encode Project Consortium *et al.* Expanded encyclopaedias of DNA elements in the human and mouse genomes. *Nature* **583**, 699-710 (2020).
2. Fantom Consortium the Riken Pmi Clst *et al.* A promoter-level mammalian expression atlas. *Nature* **507**, 462-70 (2014).
3. Kimmel, C.B., Ballard, W.W., Kimmel, S.R., Ullmann, B. & Schilling, T.F. Stages of embryonic development of the zebrafish. *Developmental Dynamics* **203**, 253-310 (1995).
4. Durinck, S. *et al.* BioMart and Bioconductor: a powerful link between biological databases and microarray data analysis. *Bioinformatics* **21**, 3439-3440 (2005).
5. Murata, M. *et al.* Detecting Expressed Genes Using CAGE. in *Transcription Factor Regulatory Networks* 67-85 (2014).
6. Takahashi, H., Lassmann, T., Murata, M. & Carninci, P. 5′ end–centered expression profiling using cap-analysis gene expression and next-generation sequencing. *Nature Protocols* **7**, 542-561 (2012).
7. Buenrostro, J.D., Giresi, P.G., Zaba, L.C., Chang, H.Y. & Greenleaf, W.J. Transposition of native chromatin for fast and sensitive epigenomic profiling of open chromatin, DNA-binding proteins and nucleosome position. *Nature Methods* **10**, 1213-1218 (2013).
8. D'Orazio, F.M. *et al.* Germ cell differentiation requires Tdrd7-dependent chromatin and transcriptome reprogramming marked by germ plasm relocalization. *Developmental Cell* **56**, 641-656.e5 (2021).

9. Díaz, N. *et al.* Chromatin conformation analysis of primary patient tissue using a low input Hi-C method. *Nature Communications* **9**(2018).
10. Acemel, R.D. *et al.* A single three-dimensional chromatin compartment in amphioxus indicates a stepwise evolution of vertebrate Hox bimodal regulation. *Nature Genetics* **48**, 336-341 (2016).
11. Kuleshov, M.V. *et al.* Enrichr: a comprehensive gene set enrichment analysis web server 2016 update. *Nucleic Acids Res* **44**, W90-7 (2016).
12. Hahne, F. & Ivanek, R. Visualizing Genomic Data Using Gviz and Bioconductor. in *Statistical Genomics* 335-351 (2016).
13. Thodberg, M., Thieffry, A., Vitting-Seerup, K., Andersson, R. & Sandelin, A. CAGEfightR: analysis of 5'-end data using R/Bioconductor. *BMC Bioinformatics* **20**, 487 (2019).
14. Fornes, O. *et al.* JASPAR 2020: update of the open-access database of transcription factor binding profiles. *Nucleic Acids Research* (2019).
15. Tan, G. & Lenhard, B. TFBSTools: an R/bioconductor package for transcription factor binding site analysis. *Bioinformatics* **32**, 1555-1556 (2016).
16. Engström, P.G., Fredman, D. & Lenhard, B. Ancora: a web resource for exploring highly conserved noncoding elements and their association with developmental regulatory genes. *Genome Biology* **9**(2008).
17. Hubisz, M.J., Pollard, K.S. & Siepel, A. PHAST and RPHAST: phylogenetic analysis with space/time models. *Briefings in Bioinformatics* **12**, 41-51 (2010).
18. Chen, Z. *et al.* De novo assembly of the goldfish (Carassius auratus) genome and the evolution of genes after whole-genome duplication. *Science Advances* **5**(2019).
19. Kohonen, T. Self-organized formation of topologically correct feature maps. *Biological Cybernetics* **43**, 59-69 (1982).
20. Wehrens, R. & Kruisselbrink, J. Flexible Self-Organizing Maps in kohonen 3.0. *Journal of Statistical Software* **87**(2018).
21. Akalin, A., Franke, V., Vlahovi ek, K., Mason, C.E. & Schubeler, D. genomation: a toolkit to summarize, annotate and visualize genomic intervals. *Bioinformatics* **31**, 1127-1129 (2014).
22. Zhang, Y. *et al.* Model-based Analysis of ChIP-Seq (MACS). *Genome Biology* **9**(2008).
23. Jiang, L. *et al.* Sperm, but Not Oocyte, DNA Methylome Is Inherited by Zebrafish Early Embryos. *Cell* **153**, 773-784 (2013).
24. Potok, M.E., Nix, D.A., Parnell, T.J. & Cairns, B.R. Reprogramming the Maternal Zebrafish Genome after Fertilization to Match the Paternal Methylation Pattern. *Cell* **153**, 759-772 (2013).
25. Bogdanović, O. *et al.* Active DNA demethylation at enhancers during the vertebrate phylotypic period. *Nature Genetics* **48**, 417-426 (2016).
26. Bolger, A.M., Lohse, M. & Usadel, B. Trimmomatic: a flexible trimmer for Illumina sequence data. *Bioinformatics* **30**, 2114-2120 (2014).
27. Chen, H., Smith, A.D. & Chen, T. WALT: fast and accurate read mapping for bisulfite sequencing. *Bioinformatics* **32**, 3507-3509 (2016).
28. Tarasov, A., Vilella, A.J., Cuppen, E., Nijman, I.J. & Prins, P. Sambamba: fast processing of NGS alignment formats. *Bioinformatics* **31**, 2032-2034 (2015).
29. Li, H. *et al.* The Sequence Alignment/Map format and SAMtools. *Bioinformatics* **25**, 2078-2079 (2009).

30. Kent, W.J., Zweig, A.S., Barber, G., Hinrichs, A.S. & Karolchik, D. BigWig and BigBed: enabling browsing of large distributed datasets. *Bioinformatics* **26**, 2204-2207 (2010).
31. Ramírez, F., Dündar, F., Diehl, S., Grüning, B.A. & Manke, T. deepTools: a flexible platform for exploring deep-sequencing data. *Nucleic Acids Research* **42**, W187-W191 (2014).
32. Quinlan, A.R. & Hall, I.M. BEDTools: a flexible suite of utilities for comparing genomic features. *Bioinformatics* **26**, 841-842 (2010).
33. McGarvey, A.C. *et al.* Single-cell-resolved dynamics of chromatin architecture delineate cell and regulatory states in zebrafish embryos. *Cell Genomics* **2**(2022).
34. Briggs, J.A. *et al.* The dynamics of gene expression in vertebrate embryogenesis at single-cell resolution. *Science* **360**(2018).
35. Lawrence, M., Gentleman, R. & Carey, V. rtracklayer: an R package for interfacing with genome browsers. *Bioinformatics* **25**, 1841-1842 (2009).
36. Kent, W.J. *et al.* The Human Genome Browser at UCSC. *Genome Research* **12**, 996-1006 (2002).
37. Nepal, C. *et al.* Dynamic regulation of the transcription initiation landscape at single nucleotide resolution during vertebrate embryogenesis. *Genome Research* **23**, 1938-1950 (2013).
38. Kruse, K., Hug, C.B. & Vaquerizas, J.M. FAN-C: a feature-rich framework for the analysis and visualisation of chromosome conformation capture data. *Genome Biology* **21**(2020).
39. Kruse, K., Hug, C.B., Hernández-Rodríguez, B. & Vaquerizas, J.M. TADtool: visual parameter identification for TAD-calling algorithms. *Bioinformatics* **32**, 3190-3192 (2016).
40. Nash, A.J. & Lenhard, B. A novel measure of non-coding genome conservation identifies genomic regulatory blocks within primates. *Bioinformatics* **35**, 2354-2361 (2019).
41. Wingett, S. *et al.* HiCUP: pipeline for mapping and processing Hi-C data. *F1000Res* **4**, 1310 (2015).
42. Langmead, B. & Salzberg, S.L. Fast gapped-read alignment with Bowtie 2. *Nat Methods* **9**, 357-9 (2012).
43. Heinz, S. *et al.* Simple combinations of lineage-determining transcription factors prime cis-regulatory elements required for macrophage and B cell identities. *Mol Cell* **38**, 576-89 (2010).
44. Gu, Z., Eils, R. & Schlesner, M. Complex heatmaps reveal patterns and correlations in multidimensional genomic data. *Bioinformatics* **32**, 2847-2849 (2016).
45. Durinck, S., Spellman, P.T., Birney, E. & Huber, W. Mapping identifiers for the integration of genomic datasets with the R/Bioconductor package biomaRt. *Nature Protocols* **4**, 1184-1191 (2009).
