# Supplementary material for: Multiomic atlas with functional stratification and developmental dynamics of zebrafish cis-regulatory elements
Source: Nat Genet. 2022 Jul 4;54(7):1037–50. doi: 10.1038/s41588-022-01089-w (PMC9279159; doi:10.1038/s41588-022-01089-w)
Supplement: Supplementary file 2 [file 41588_2022_1089_MOESM2_ESM.pdf]

nature portfolio

Corresponding author(s): Dr. Ferenc Mueller
Dr. Boris Lenhard
Dr. Carsten O. Daub

Last updated by author(s): 2022-13-04

Reporting Summary

Nature Portfolio wishes to improve the reproducibility of the work that we publish. This form provides structure for consistency and transparency in reporting. For further information on Nature Portfolio policies, see our Editorial Policies and the Editorial Policy Checklist.

Statistics

For all statistical analyses, confirm that the following items are present in the figure legend, table legend, main text, or Methods section.

n/a	Confirmed	
☐	☒	The exact sample size (*n*) for each experimental group/condition, given as a discrete number and unit of measurement
☐	☒	A statement on whether measurements were taken from distinct samples or whether the same sample was measured repeatedly
☐	☒	The statistical test(s) used AND whether they are one- or two-sided<br>*Only common tests should be described solely by name; describe more complex techniques in the Methods section.*
☐	☒	A description of all covariates tested
☐	☒	A description of any assumptions or corrections, such as tests of normality and adjustment for multiple comparisons
☐	☒	A full description of the statistical parameters including central tendency (e.g. means) or other basic estimates (e.g. regression coefficient) AND variation (e.g. standard deviation) or associated estimates of uncertainty (e.g. confidence intervals)
☐	☒	For null hypothesis testing, the test statistic (e.g. *F*, *t*, *r*) with confidence intervals, effect sizes, degrees of freedom and *P* value noted<br>*Give P values as exact values whenever suitable.*
☒	☐	For Bayesian analysis, information on the choice of priors and Markov chain Monte Carlo settings
☐	☒	For hierarchical and complex designs, identification of the appropriate level for tests and full reporting of outcomes
☐	☒	Estimates of effect sizes (e.g. Cohen's *d*, Pearson's *r*), indicating how they were calculated

Our web collection on statistics for biologists contains articles on many of the points above.

Software and code

Policy information about availability of computer code

Data collection

Data collection: DANIO-CODE DCC is based on *-DCC (https://gitlab.com/danio-code/public/dcc).
Data processing: The raw sequencing files were mapped using the DANIO-CODE pipelines (https://gitlab.com/danio-code,v1.1).
The genome track visualisations are based on WahsU Epigenome Browser and the UCSC genome browser. Further visualisation of chromatin marks and gene tracks was done using Gviz (https://github.com/ivanek/Gviz,v1.38.4).
CAGE data were further processed with CAGEr (https://bioconductor.org/packages/CAGEr/, v1.20). The enhancer were called using CageFightR (https://www.bioconductor.org/packages/CAGEfightR/,v1.8.0)
The 4C tracks were mapped using bowtie1 (https://github.com/BenLangmead/bowtie,v1.2.3).
The BS-seq were processed using Trimmomatic (https://github.com/usadellab/Trimmomatic,v0.38), sambamba (https://github.com/biod/sambamba,v0.6.8), samtools (https://github.com/samtools/samtools,v1.9), picard (https://github.com/broadinstitute/picard,v2.18.25), MethylDackel (https://github.com/dpryan79/MethylDackel,v0.3.0), UCSC kentUtils (https://github.com/ENCODE-DCC/kentUtils,v302.1 kentUtils 2014-11-11), deepTools (https://github.com/deeptools/deepTools,v3.1.1), and BEDTools (https://github.com/arq5x/bedtools2,v2.27.1)
RNA-Seq and aligned to GRCz10 using STAR aligner (https://github.com/alexdobin/STAR, v. 2.5.1b91).
StringTie was used to call transcripts (https://ccb.jhu.edu/software/stringtie/, v.1.33b92)
RNA-seq transcript were assembled using TACO (https://github.com/tacorna/taco,v0.7.3) and quantified using Salmon (https://github.com/COMBINE-lab/salmon, v0.11.2).
The genomic regions were segmented and annotated with ChromHMM (https://github.com/jernst98/ChromHMM, v1.12).
The nucleosome positions were calculated by NucleoATAC (https://github.com/GreenleafLab/NucleoATAC, v0.3.4).
CTCF binding sites were identified using TFBSTools (https://www.bioconductor.org/packages/TFBSTools/, v1.28)
danRer10 to danRer 11 lift over was performed using the rtracklayer package (https://bioconductor.org/packages/rtracklayer/,v1.54.0)
Self-organizing-maps were generated using the kohonen package (https://cran.r-project.org/web/packages/kohonen,v.3.0.8)
The genomation package was used to calculate the mean nucleosome signal of the cPADRES as well as to visualise the ATAC and ChIP-seq signals (https://bioconductor.org/packages/genomation/,v1.26.0) and visualised using the uwot (https://cran.r-project.org/web/packages/uwot/, v0.1.10).

Peak summits were defined using MACS2 (https://github.com/macs3-project/MACS, v2.2.7.1).
The Hi-C data was processed using HiCUP (https://github.com/StevenWingett/HiCUP,v0.6.0) and mapped using Bowtie2 (https://github.com/BenLangmead/bowtie2,v2.3.4.1). The aligned data were further processed using HOMER (http://homer.ucsd.edu/homer,v4.11).
Hi-C matrices were processed using FAN-C (https://github.com/vaquerizaslab/fanc,v0.9.0). TADs were called using TADtool (https://github.com/vaquerizaslab/tadtool, v.0.76). The heatmap for CAGE promoters in TADs were generated by the ComplexHeatmap package (https://www.bioconductor.org/packages/ComplexHeatmap/, v0.75).
The Enhancer ensembles were detected using the ROSE algorithm (https://bitbucket.org/young_computation/rose, commit feb35cb1d9556a76f8ac1f51521539bb30651343). The H3K27ac signal across TADs was visualised using the heatmaps package (https://bioconductor.org/packages/heatmaps/, v1.18.0)
Motif hits for the conservation analysis were computed using seqPattern (https://bioconductor.org/packages/seqPattern, v1.26.0).

Data analysis

The code used for data analysis is available in the following GitHub repository:
https://github.com/DANIO-CODE/DANIO-CODE_Data_analysis

For manuscripts utilizing custom algorithms or software that are central to the research but not yet described in published literature, software must be made available to editors and reviewers. We strongly encourage code deposition in a community repository (e.g. GitHub). See the Nature Portfolio guidelines for submitting code & software for further information.

Data

Policy information about availability of data

All manuscripts must include a data availability statement. This statement should provide the following information, where applicable:

- Accession codes, unique identifiers, or web links for publicly available datasets
- A description of any restrictions on data availability
- For clinical datasets or third party data, please ensure that the statement adheres to our policy

All the data are available on DANIO-CODE Data Coordination Center (https://danio-code.zfin.org). The previously unpublished data are available under the following IDs: DCD000519SR, DCD000518SR, DCD000514SR, DCD000493SR, DCD000490SR, DCD000244SR
The raw sequencing data produced for this study are available on the European Bioinformatics Institute (EBI) European Nucleotide Archive (ENA) under study numbers PRJNA824720, PRJNA821001, PRJNA821088, PRJNA821148, and PRJNA821034.

Field-specific reporting

Please select the one below that is the best fit for your research. If you are not sure, read the appropriate sections before making your selection.

☒ Life sciences ☐ Behavioural & social sciences ☐ Ecological, evolutionary & environmental sciences

For a reference copy of the document with all sections, see nature.com/documents/nr-reporting-summary-flat.pdf

Life sciences study design

All studies must disclose on these points even when the disclosure is negative.

Sample size

Sample size was determined by availability of genomics datasets which were collected from public databases.

Data exclusions

Genomics data were excluded which did not meet the quality control criteria (as set by ENCODE criteria) and which when compared by PCA was identified as technical outlier.

Replication

Genomics data were used where at least 2 replicates were available. The complete datasets consists of two parts: 1. publicly available datasets collected and re-processed in a standardised way as a part of this study. The replication in those cases were constrained to data availability. All data used in the analysis of this paper passed the quality control. 2. Newly generated data for this study: All of the newly generated data for this study passed the quality control and reproducibility controls, except for 2 H3K4me3 Long-Pec samples, which subsequently haven't been used in further analyses.
Summary of all datasets and their numbers is available in Figure 1. Details about all datasets, as well if they were used in the analysis for this paper are available as Supplementary Table 1.

Randomization

Not applicable due to the nature of analyses of genomics datasets (there was no case/control experimental design in this study). The zebrafish embryos were collected by standard laboratory procedure described in the Supplementary methods section.

Blinding

Not applicable due to the nature of analyses of genomics datasets (there was no case/control experimental design in this study). The zebrafish embryos were collected by standard laboratory procedure described in the Supplementary methods section..

Reporting for specific materials, systems and methods

We require information from authors about some types of materials, experimental systems and methods used in many studies. Here, indicate whether each material, system or method listed is relevant to your study. If you are not sure if a list item applies to your research, read the appropriate section before selecting a response.

Materials & experimental systems

n/a	Involved in the study
☐	☒ Antibodies
☒	☐ Eukaryotic cell lines
☒	☐ Palaeontology and archaeology
☐	☒ Animals and other organisms
☒	☐ Human research participants
☒	☐ Clinical data
☒	☐ Dual use research of concern

Methods

n/a	Involved in the study
☐	☒ ChIP-seq
☒	☐ Flow cytometry
☒	☐ MRI-based neuroimaging

Antibodies

Antibodies used

Polyclonal rabbit anti-H3K4me3 from Diagenode, Cat-No. C15410003
Polyclonal rabbit anti- H3K27ac from abcam, Cat-No. ab4729
Polyclonal rabbit anti-H3K4me1 from abcam, Cat-No. ab8895
Polyclonal rabbit anti-H3K27me3 from Diagenode, Cat-No. C15410069
Polyclonal rabbit anti-H3K36me3 from abcam, Cat-No. ab9050
Polyclonal rabbit anti- Pol II (phospho S5) from abcam, Cat-No. ab5131
Polyclonal rabbit anti-H2A.Z from abcam, Cat-No. ab4174

Validation

Polyclonal rabbit anti-H3K4me3 from Diagenode, Cat-No. C15410003 was validated for ChIP-qPCR by the manufacturer using human K562 cells. Species reactivity: Human, mouse, zebrafish, trout, Daphnia, Arabidopsis, rice, tomato, maize, poplar, silena latifolia.
Polyclonal rabbit anti- H3K27ac from abcam, Cat-No. ab4729 was validated for X-ChIP by the manufacturer using HeLa cells. Predicted reactivity with Rat, Chicken, Xenopus laevis, Arabidopsis thaliana, Drosophila melanogaster, Monkey, Zebrafish, Plasmodium falciparum, Rice, Cyanidioschyzon merolae.
Polyclonal rabbit anti-H3K4me1 from abcam, Cat-No. ab8895 was validated for X-ChIP by the manufacturer using U-2 OS cells and previously reported to cross-react with zebrafish samples.
Polyclonal rabbit anti-H3K27me3 from Diagenode, Cat-No. C15410069 was validated for ChIP by the manufacturer using K562 cells. Species reactivity: Human, mouse, rat, pig, zebrafish, Drosophila, Schistosoma, Arabidopsis, cow
Polyclonal rabbit anti-H3K36me3 from abcam, Cat-No. ab9050 was validated for X-ChIP by the manufacturer using U-2 OS cells. Predicted reactivity: Mouse, Rat, Saccharomyces cerevisiae, Xenopus laevis, Arabidopsis thaliana, Caenorhabditis elegans, Drosophila melanogaster, Plants, Schizosaccharomyces pombe, Zebrafish, Silk worm, Rice, Xenopus tropicalis, Trypanosoma brucei.
Polyclonal rabbit anti- Pol II (phosphor S5) from abcam, Cat-No. ab5131 was validated for X-ChIP by the manufacturer using U-2 OS cells. Predicted reactivity: Mouse, Rat, Saccharomyces cerevisiae, Xenopus laevis, Arabidopsis thaliana, Caenorhabditis elegans, Drosophila melanogaster, Schizosaccharomyces pombe, Zebrafish, a wide range of other species.
Polyclonal rabbit anti-H2A.Z from abcam, Cat-No. ab4174 was validated for X-ChIP by the manufacturer using HeLa cells. Predicted reactivity: Sheep, Xenopus laevis, Arabidopsis thaliana, Zebrafish.

Animals and other organisms

Policy information about studies involving animals; ARRIVE guidelines recommended for reporting animal research

Laboratory animals

In this study, 6-18 month old zebrafish males and females belonging to the Ab and Buc-GFP strains were exclusively used for natural breeding. Fish were maintained in a designated facility according to the UK Home Office regulations and UK Animals (Scientific Procedures) Act 1986. All animals were kept in a recirculating system (ZebTEC, Tecniplast) in a 10-hour dark, 14-hour light photoperiod at 26°C and fed 3 times daily.
Zebrafish embryos were obtained by crossing one male and one female (separated by a divider) in a specifically designed crossing cage in the evening. On the next morning, dividers were removed, and embryos produced by natural spawning were collected shortly (5-10 min) after laying by using a small net.

Wild animals

No wild animals were used in this study.

Field-collected samples

No field collected samples were used in this study.

Ethics oversight

Wild type (AB) and Tg(Buc-GFP) strains were maintained in a designated facility according to the UK Home Office regulations and UK Animals (Scientific Procedures) Act 1986. Fish were kept in a recirculating system (ZebTEC, Tecniplast) in a 10-hour dark, 14-hour light photoperiod at 26°C and fed 3 times daily.
Zebrafish experiments were restricted to embryonic stages and adults were only used for natural breeding under the project licences assigned to the universities involved in this project. Zebrafish embryos were obtained by crossing one male and one female (separated by a divider) in a specifically designed crossing cage in the evening. On the next morning, dividers were removed, and embryos produced by natural spawning were collected shortly (5-10 min) after laying by using a small net.

Note that full information on the approval of the study protocol must also be provided in the manuscript.

ChIP-seq

Data deposition

☒ Confirm that both raw and final processed data have been deposited in a public database such as GEO.

☒ Confirm that you have deposited or provided access to graph files (e.g. BED files) for the called peaks.

Data access links<br>*May remain private before publication.*	https://danio-code.zfin.org/dataExport/daniocode/detailSeries/518/
Files in database submission	Along with raw fastq reads, for each sample a signal (p-value of signal over control) file in bigWig format is provided, along with peaks in narrowPeak file format.
Genome browser session<br>(e.g. UCSC)	Processed data can be viewed in the DANIO-CODE Track Hub, listed in the UCSC Genome browser public Track Hubs, or uploadable through the link: https://danio-code.zfin.org/trackhub/DANIO-CODE.hub.txt

Methodology

Replicates	5 - 9 somites samples and Long-Pec samples (H3K27ac, H3K4me1, and H3K27me3) were done in 1 replicate, as these samples were already available in 1 replicate from before.<br>Long-pec H2AZ, PolII, H3K4me3 were done in 2 replicates.
Sequencing depth	All samples were 75bp paired-end datasets.<br>The sequencing depth for each sample after optical duplicates removal:<br>5-9 somites H3K27me3 26938306<br>H3K4me1 14905594<br>H3K36ne3 43630140<br>Long-pec H2AZ rep1 18712668<br>rep2 36137656<br>H3K4me3 rep1 13346874<br>rep2 21845236<br>PolII rep1 41158384<br>rep2 16735286<br>H3K27ac 58048920<br>H3K27me3 59785820<br>H3K4me1 53491012
Antibodies	Polyclonal rabbit anti-H3K4me3 from Diagenode, Cat-No. C15410003<br>Polyclonal rabbit anti- H3K27ac from abcam, Cat-No. ab4729<br>Polyclonal rabbit anti-H3K4me1 from abcam, Cat-No. ab8895<br>Polyclonal rabbit anti-H3K27me3 from Diagenode, Cat-No. C15410069<br>Polyclonal rabbit anti-H3K36me3 from abcam, Cat-No. ab9050<br>Polyclonal rabbit anti- Pol II (phospho S5) from abcam, Cat-No. ab5131<br>Polyclonal rabbit anti-H2A.Z from abcam, Cat-No. ab4174
Peak calling parameters	Program: macs2 callpeak<br>Parameters: -f BED -g 1371719383 -p 0.01 --nomodel --shift 0 --extsize 205 --keep-dup all -B --SPMR
Data quality	For the following samples fraction of reads in peaks was used as a measure of quality:<br>5-9 somites H3K27me3 0.2323<br>H3K4me1 0.3395<br>H3K36ne3 0.2524<br>Long-pec H2AZ rep1 0.2539<br>rep2 0.2539<br>H3K4me3 rep1 0.0071 - not used in analysis<br>rep2 0.0071 - not used in analysis<br>PolII rep1 0.0256<br>rep2 0.0256<br>For the following samples, the proportion of peaks overlapping in pseudo-replicates was used as a method of quality check:<br>Long-pec H3K27ac 0.5935<br>H3K27me3 0.2562<br>H3K4me1 0.5098
Software	The code and detail for ChIP-seq processing are available on: https://gitlab.com/danio-code/DANIO-CODE_ChIP-seq
